# Impact of duration and magnitude of raised intracranial pressure on outcome after severe traumatic brain injury: A CENTER-TBI high-resolution group study

**DOI:** 10.1371/journal.pone.0243427

**Published:** 2020-12-14

**Authors:** Cecilia AI Åkerlund, Joseph Donnelly, Frederick A. Zeiler, Raimund Helbok, Anders Holst, Manuel Cabeleira, Fabian Güiza, Geert Meyfroidt, Marek Czosnyka, Peter Smielewski, Nino Stocchetti, Ari Ercole, David W. Nelson, the CENTER-TBI High Resolution ICU Sub-Study Participants and Investigators

**Affiliations:** 1 Department of Physiology and Pharmacology, Section of Perioperative Medicine and Intensive Care, Karolinska Institutet, Stockholm, Sweden; 2 School of Computer Science and Communication, KTH Royal Institute of Technology, Stockholm, Sweden; 3 Clinical Neuroscience, University of Cambridge, Cambridge, United Kingdom; 4 Section of Neurosurgery, Department of Surgery, Rady Faculty of Health Sciences, University of Manitoba, Winnipeg, Manitoba, Canada; 5 Department of Anatomy and Cell Science, Rady Faculty of Health Sciences, University of Manitoba, Winnipeg, Manitoba, Canada; 6 Biomedical Engineering, Faculty of Engineering, University of Manitoba, Winnipeg, Manitoba, Canada; 7 Centre on Aging, University of Manitoba, Winnipeg, Manitoba, Canada; 8 Division of Anaesthesia, University of Cambridge, Cambridge, United Kingdom; 9 Department of Neurology, Neurological Intensive Care Unit, Medical University of Innsbruck, Innsbruck, Austria; 10 Department and Laboratory of Intensive Care Medicine, University Hospitals Leuven and KU Leuven, Leuven, Belgium; 11 Institute of Electronic Systems, Warsaw University of Technolology, Warszawa, Poland; 12 Department of Pathophysiology and Transplants, University of Milan, and Neuroscience Intensive Care Unit, Fondazione IRCCS Cà Granda Ospedale Maggiore Policlinico, Milan, Italy; University of Florida, UNITED STATES

## Abstract

Magnitude of intracranial pressure (ICP) elevations and their duration have been associated with worse outcomes in patients with traumatic brain injuries (TBI), however published thresholds for injury vary and uncertainty about these levels has received relatively little attention. In this study, we have analyzed high-resolution ICP monitoring data in 227 adult patients in the CENTER-TBI dataset. Our aim was to identify thresholds of ICP intensity and duration associated with worse outcome, and to evaluate the uncertainty in any such thresholds. We present ICP intensity and duration plots to visualize the relationship between ICP events and outcome. We also introduced a novel bootstrap technique to evaluate uncertainty of the equipoise line. We found that an intensity threshold of 18 ± 4 mmHg (2 standard deviations) was associated with worse outcomes in this cohort. In contrast, the uncertainty in what duration is associated with harm was larger, and safe durations were found to be population dependent. The pressure and time dose (PTD) was also calculated as area under the curve above thresholds of ICP. A relationship between PTD and mortality could be established, as well as for unfavourable outcome. This relationship remained valid for mortality but not unfavourable outcome after adjusting for IMPACT core variables and maximum therapy intensity level. Importantly, during periods of impaired autoregulation (defined as pressure reactivity index (PRx)>0.3) ICP events were associated with worse outcomes for nearly all durations and ICP levels in this cohort and there was a stronger relationship between outcome and PTD. Whilst caution should be exercised in ascribing causation in observational analyses, these results suggest intracranial hypertension is poorly tolerated in the presence of impaired autoregulation. ICP level guidelines may need to be revised in the future taking into account cerebrovascular autoregulation status considered jointly with ICP levels.

## Introduction

Traumatic brain injury (TBI) is a major cause of worldwide mortality and morbidity [1]. A key goal of the neurointensive care of the most severely injured patients is to minimize secondary injury through interventions based on the close monitoring of intracranial and systemic physiology.

One of the most important physiological parameters in modern neurocritical care is intracranial pressure (ICP). Supported by a study on the TBI database in Cambridge, UK [2] the Brain Trauma Foundation (BTF) guidelines state that ICPs above 22 mmHg should be treated as this is associated with increased mortality [3], and in Europe there is a general consensus that ICP levels above 20 mmHg should be actively managed [4]. Despite this general consensus some studies have shed doubt on the efficacy of ICP monitoring itself [5–8] and question the validity of treating such fixed values. Indeed, without effective management strategies, monitoring by itself cannot improve outcome and heterogeneous treatment strategies may contribute as to explain a lack of established efficacy of monitoring. In particular absolute ‘safe’ levels of ICP have not conclusively been shown, however some attempts have been noted [9]. Additionally, there is no general consensus on what durations of increased ICP levels might be tolerated before harm is caused.

Automatic recording of physiological parameters has been shown to have advantages over manual detection of secondary insults in brain injuries [10, 11]. Continuous recording has made it possible to study the time and pressure dose of ICP in more detail. There has been increasing interest in the impact of the duration of elevated intracranial pressure, both in TBI, and in patients with other than acute brain syndromes. In two single-center studies [12, 13], with 93 and 60 TBI patients respectively, an association was found between an increased pressure-time dose of ICP and poor outcome at 6 months post injury. Similar results have been observed in a cohort of patients with spontaneous subarachnoid haemorrhage, although the underlying pathophysiology is likely to be different from TBI [14].

An important contribution to understanding the impact of insult duration on outcome was the insult intensity / duration plots described by Güiza et al [15] which correlated the number of events above increasing thresholds of pressure and time with outcome, visualizing the results on a colour-coded grid. The intensity/duration plot has shed important light on the relation between ICP events and their duration and outcome. Donnelly et al [16] produced a similar plot albeit with different cut-offs and using data from another cohort of TBI patients. The difference in results between the previous studies implies that ICP tolerability levels might not be universal, but cohort dependent and that the results are associated with some degree of uncertainty. As this uncertainty has not yet been investigated, and these types of plots may be widely used to identify perceived safe levels and durations of raised ICP, it is essential to investigate and establish the certainty of these plots. The aims of this study are thus to investigate the impact of ICP intensity and duration on outcome in the large multi-center cohort in the CENTER-TBI study [17, 18], to examine the impact of cerebrovascular autoregulation status on ICP tolerability and to quantify the certainty/uncertainty of identifiable ICP injury thresholds.

## Materials and methods

High-frequency ICP (up to 500 Hz) and arterial blood pressure signals were recorded in 273 patients from 20 different sites participating in the European multi-center study CENTER-TBI, using the software ICM+ (Cambridge Enterprise Ltd, University of Cambridge, UK, versions 8.4.4.4 to 8.5.5.1), or a combination of ICM+ and CNS Monitor (Moberg Research Inc, Ambler, PA, USA), between January 2015 and March 2018. Pressure reactivity Index (PRx), the moving Pearson correlation between ICP and arterial blood pressure, was calculated using standard methodology in ICM+ [19, 20]. Data for the CENTER-TBI study was collected through the Quesgen e-CRF (Quesgen Systems Inc, USA), hosted on the INCF platform and extracted via the INCF Neurobot tool (INCF, Sweden). Version 2.1 of the CENTER-TBI dataset was used in this manuscript.

All patients met the general inclusion criteria for CENTER-TBI (Clinical diagnosis of TBI, clinical indication for CT scan and presentation within 24 hours of injury) and were admitted directly from the ER to the ICU [17]. This study was approved by the CENTER-TBI management committee. The CENTER-TBI study was conducted in accordance with all relevant laws of the European Union if directly applicable or of direct effect and all relevant laws of the country where the Recruiting sites were located, including but not limited to, the relevant privacy and data protection laws and regulations (the “Privacy Law”), the relevant laws and regulations on the use of human materials, and all relevant guidance relating to clinical studies from time to time in force including, but not limited to, the ICH Harmonised Tripartite Guideline for Good Clinical Practice (CPMP/ICH/135/95) (“ICH GCP”) and the World Medical Association Declaration of Helsinki. Written or oral Informed Consent by the patients or next of kin was obtained, accordingly to the local legislations, for all patients recruited in the Core Dataset of CENTER-TBI and documented in the electronic case report form. In case of oral consent, a written confirmation was requested.

Ethical approval was obtained for each recruiting site. The list of sites, Ethical Committees, approval numbers and approval dates are available online [21] and ethical approval numbers for sites having recruited patients to the high-resolution sub-study of CENTER-TBI is listed in S1 Appendix.

### Data preparation

One-minute averages of ICP data were calculated from 10-second summaries. Data from patients with ventriculostomies was included: External ventricular drains (EVD) were confirmed to have been closed throughout the monitoring period by manual inspection of the ICP waveforms. Data from the day of trauma through day 7 were used for the calculation of ICP burden, based on previous results that mean ICP differs between survivors and non-survivors only the first 7 days post injury [22].

The Glasgow Outcome Scale Extended (GOS-E) 6 months post injury was used as outcome measure, where 1 indicates death and 8 good recovery without disability. If GOS-E scores at 6 months were missing, a derived GOS-E score was used. A multi-state model created centrally in CENTER-TBI was used if at least one GOS-E value was present outside the pre-specified time window for 6 months. If GOS-E score was missing and could not be imputed, the patient was removed from the final analysis.

Forty-six patients were excluded due to monitoring time shorter than one day (*n* = 8), missing GOS-E score at 6 months (*n* = 30) or missing baseline data (*n* = 8), leaving 227 patients for the final analysis.

### Correlations between number of events above thresholds of pressure and time and outcome

The correlation between number of insults above thresholds of ICP and duration was calculated using the insult intensity/duration plot method described previously [15]: The correlations are presented in a grid where each pixel is represented by a colour (blue = positive i.e. better outcome, red = negative, i.e. worse outcome). It corresponds to the Pearson correlation coefficient between mean number of events and GOS-E score for each position on the plot. Each position on the plot represents events above the given ICP level and of a duration. Thresholds of ICP between 10 and 40 mmHg and duration from 5 to 360 minutes were used generating a grid of 11,036 pixels.

PRx, the moving Pearson correlation between ICP and arterial blood pressure, was provided in the measurement files. By averaging PRx for each event, all events were classified into either impaired (PRx > +0.3) or intact (PRx <= 0.3) autoregulation. The cut-off of PRx +0.3 was chosen as threshold, as it previously has been suggested to be associated with worse outcome [2, 23, 24]. Correlations of events with either impaired or intact autoregulation were represented in separate grids. Additionally, we expanded this method as to investigate uncertainty and variability of the results with variations in patient cohorts. This was done using bootstrapping with replacement, generating 1,000 different cohorts. This is a technique where new cohorts are generated by randomly selecting 227 patients. The replacement condition implies that any patient can occur more than once in each sampled cohort. By averaging and calculating standard deviations of the correlations at each grid point, the stability and uncertainty and of the equipoise lines were investigated. By averaging the bootstrapped correlations, a mean transition line was created. Standard deviations of the correlations at each grid point were also calculated, and lines representing correlations +-2 standard deviations from the mean transition line were created.

All analyses were performed using R version 1.1.453 [25].

### Pressure and time dose of ICP (PTD)

**Fig 1:**
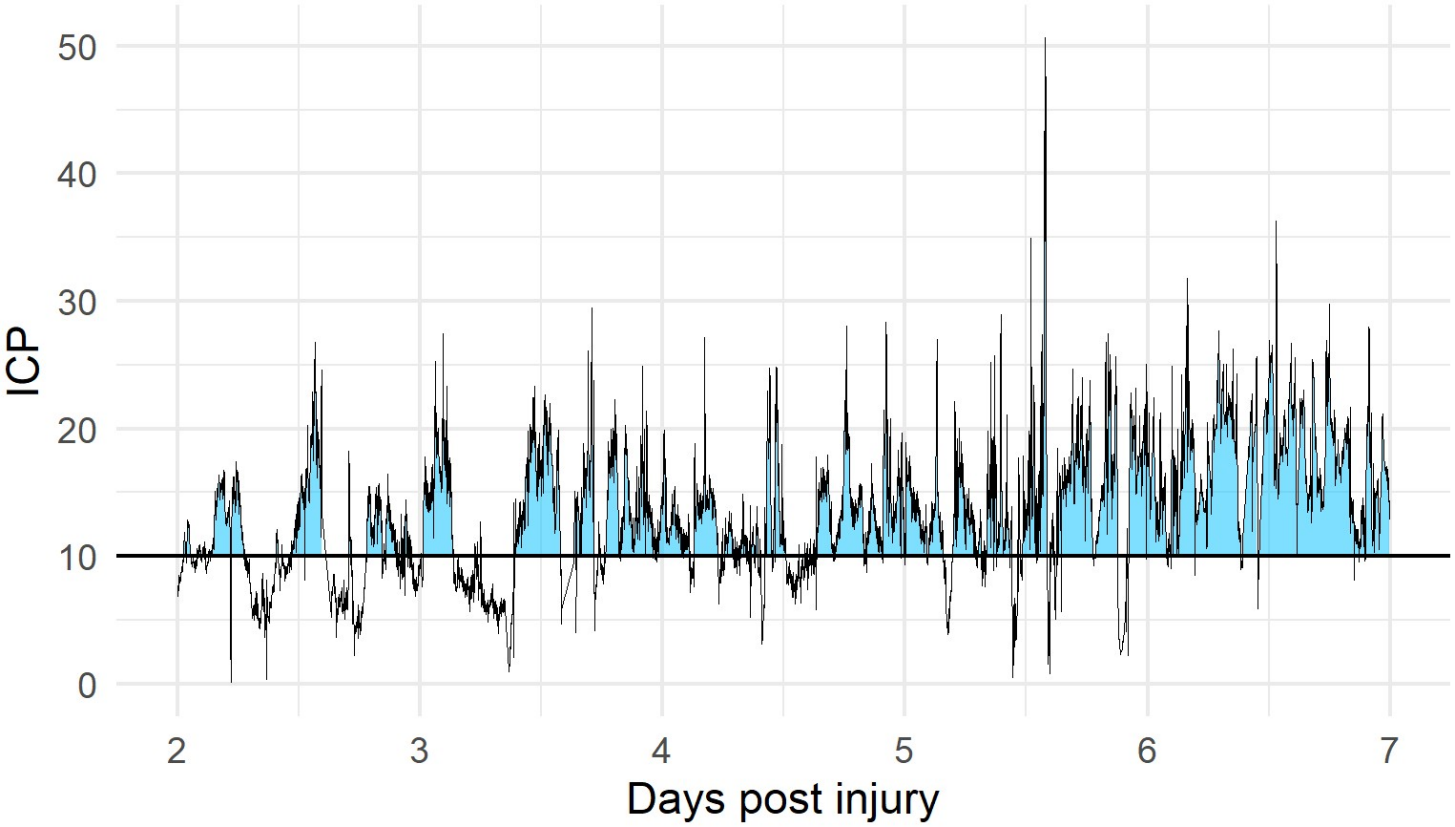
Calculation of AUC of ICP over time. An example of how ICP dose as pressure times time dose is represented from a representative patient. The blue-coloured area is the AUC (i.e. the ICP dose) above threshold ICP = 10 mmHg and represents the PTD10.

In addition to the intensity / duration plots, we investigated the pressure and time dose of ICP (PTD) as a simple alternative measure of insult severity. The PTD was calculated as the area under the curve above thresholds of ICP from 0 to 40 mmHg, as illustrated in Fig 1. Mean doses were calculated for patients with unfavourable/favourable outcome as well as for patients who were dead or alive within 6 months post injury. PTD for intact and impaired autoregulation respectively was also calculated.

GOS-E score 5 to 8 was defined as favourable and 1 to 4 as unfavourable outcome. Comparisons of distributions of PTD between groups were performed using the non-parametric Kolmogorov-Smirnov test. A threshold of 0.05 was chosen for statistical significance.

To investigate the relationship between ICP event-burden and PTD towards outcome, we performed multivariable regression analyses, adjusting for known covariates including the IMPACT core variables age, GCS motor score and pupil reactivity [26, 27] and maximum daily therapy intensity level (TIL) score.

### Time spent above transition line

To investigate the impact of co-variates on the time spent in areas of the grid with correlation to worse outcome, the time above the transition line was calculated. This was done by first determining the duration of all intensity thresholds at the transition line. For each patient and intensity threshold, the durations of events above this threshold was calculated. If the duration was longer than the duration threshold for that intensity threshold, all ICP values in that episode was regarded to be above the transition line. The duration of all ICP values above the transition line was summarized and divided by total monitoring time.

## Results

### Patient characteristics

**Fig 2:**
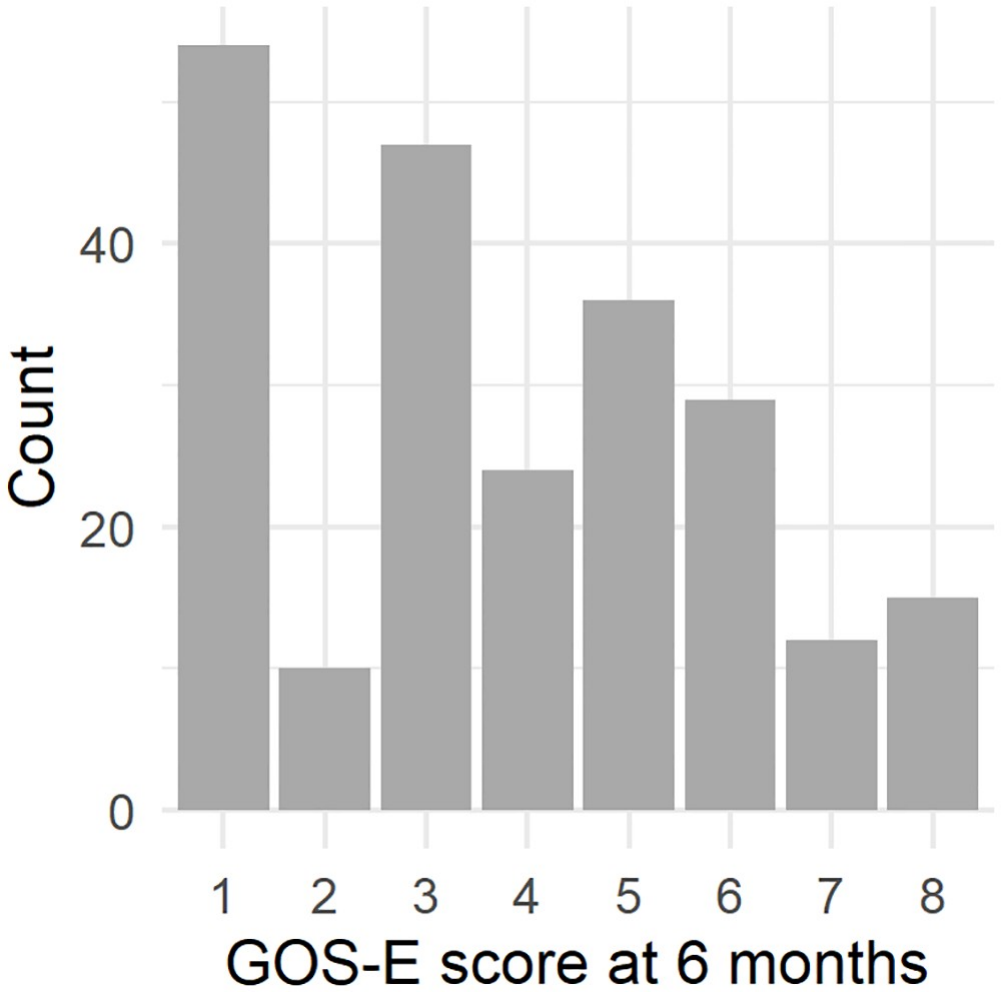
Distribution of GOS-E score at 6 months.

227 patients with high-resolution ICP measurement for more than one day, over 18 years old and with 6 month GOS-E were included in the final analysis. As presented in Table 1, our cohort consisted of 79% males with a median age of 51 years (IQR 32–64), and 50.2% had no comorbidities at time of injury (ASA class 1), indicating a fairly healthy population before injury. With a median pre-ICU Glasgow Coma Score (GCS) of 6 (IQR 3–10), the cohort can be classified as moderate to severe TBI. 53 patients (23%) underwent a decompressive craniectomy. The median GOS-E at 6 months post injury was 4 (IQR 2–5), Fig 2.

**Table 1:** Characteristics of the cohort.

**Demographic characteristics**	
Age (years)	51 (32–64)
Sex	
Female	48 (21.1)
Male	179 (78.9)
**Pre-injury health status**	
ASA-PS classification	
1	109 (50.2)
2	79 (36.4)
3	28 (12.9)
4	1 (0.5)
**Cause of injury and injury severity**	
Cause of injury	
Road traffic incident	94 (41.4)
Incidental fall	83 (36.6)
Other non-intentional injury	9 (4)
Violence/Assault	18 (7.9)
Suicide attempt	3 (1.3)
Unknown	11 (4.8)
Other	9 (4)
ISS	
Total	34 (25–43)
Highest Extracranial	9 (0–16)
Highest Head/Brain/Cervical	25 (25–25)
**Clinical presentation**	
GCS (best pre-hospital)	
Motor score	4 (1–5)
Total score	6 (3–10)
Pupillary reactivity (at baseline)	
Both reacting	152 (72.7)
One reacting	16 (7.7)
None reacting	41 (19.6)
Hypoxia (pre-ICU admission)	
No	160 (81.6)
Definite	19 (9.7)
Suspect	17 (8.7)
Hypotension (pre-ICU admission)	
No	171 (86.8)
Definite	18 (9.1)
Suspect	8 (4.1)
**CT characteristics**	
Rotterdam CT Score	4 (3–5)
Contusion	151 (74.4)
Cisternal compression	94 (46.3)
Skull fracture	129 (63.5)
Midline shift > 5 mm	67 (33)
Mass lesions > 25 ml	106 (52.2)
tSAH	175 (86.2)
EDH	45 (22.2)
aSDH	127 (62.6)
cSDH	27 (13.3)
IVH	77 (37.9)
**Other characteristics**	
Hypoxia (during hospital stay)	
No	156 (69.3)
Single episode, short duration	56 (24.9)
Multiple episodes or prolonged duration	13 (5.8)
Hypotension (during hospital stay)	
No	136 (60.4)
Single episode, short duration	61 (27.1)
Multiple episodes or prolonged duration	28 (12.4)
Type of ICP device	
Ventricular	18 (7.9)
Ventricular + inbuilt sensor	5 (2.2)
Parenchymal	191 (84.1)
Other	13 (5.7)
Decompressive craniectomy	53 (23.3)
Length of stay, days	23.73 (11.9–46.7)
Length of stay in ICU, days	13.52 (8.7–20.1)
Monitoring time, days	5.18 (3.7–7.2)
Mean ICP, mmHg	12.62 (9.4–15.4)
Mean body temperature, °C	37.07 (36.7–37.4)
Mean CPP, mmHg	70.99 (65.3–77.1)
Sodium day 2 post injury (mmol/L)	142 (139–146)
**Outcome**	
GOS-E at 6 months	
1	54 (23.8)
2	10 (4.4)
3	47 (20.7)
4	24 (10.6)
5	36 (15.9)
6	29 (12.8)
7	12 (5.3)
8	15 (6.6)

Data are median (IQR) or n (%). ASA-PS classification: American society of anesthesiologists physical status classification, ISS: Injury Severity Score, GCS: Glasgow Coma Scale, ICU: Intensive care unit, Rotterdam CT Score: a score describing the severity of findings on a CT scan, CT: Computed tomography, tSAH: Traumatic subarachnoidal haemorrhage, EDH: Epidural hematoma, aSDH: Acute subdural hematoma, cSDH: Chronic subdural hematoma, IVH: Intra-ventricular haemorrhage, ICP: Intracranial pressure, CPP: Cerebral perfusion pressure, GOS-E: Glasgow Outcome Scale extended.

### Correlations between number of insults and outcome

**Fig 3:**
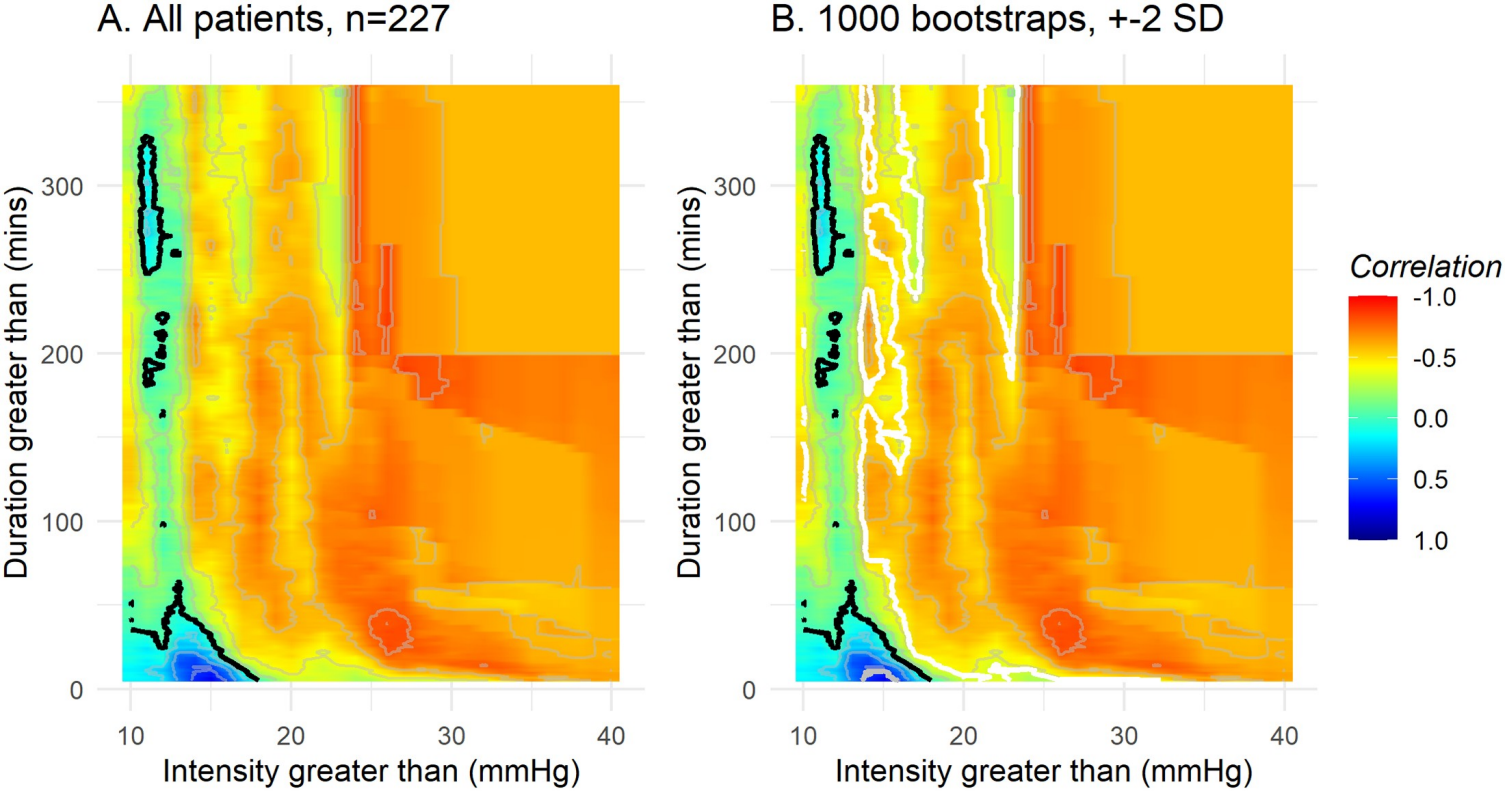
Correlation between number of events above thresholds of intracranial pressure and durations, and outcome (GOS-E score). Red indicates that ICP events are correlated to worse outcome at that specific ICP level and event duration on the map. **A.** The black line represents the transition line, where there is no correlation between number of events above threshold and outcome. **B.** The black line represents the mean transition line of 1000 bootstraps. The white lines represent the mean transition line +2 SD, while the grey line represents the mean transition line -2 SD. Above, and to the right, of the white line, there is a high degree of statistical certainty of events being associated with worse outcome, whereas below the grey line, the statistical certainty is high that events are not associated with harm.

The correlation between number of events against ICP and duration for the high-resolution cohort is presented in Fig 3A. A black transition curve divides the surface into two areas: A small blue area in the bottom left corner where number of events more frequently occur in patients with better outcome and a large red-orange area where number of events are associated with worse outcome. The transition curve represents a no correlation region between number of events and outcome.

We investigated the stability of the results by applying bootstrapping with replacement to create 1,000 different populations of 227 patients (same sample size as our cohort) to give the population dependent variability of the transition line (corresponding to correlation coefficient 0 towards GOS-E). Ten randomly selected bootstrapped correlation plots are presented in S1 Fig to illustrate how the results are affected by different cohort constitutions. Mean correlations, with the mean transition line in black (worse vs. better outcome), plus/minus two standard deviations (white), are presented in Fig 3B.

**Fig 4:**
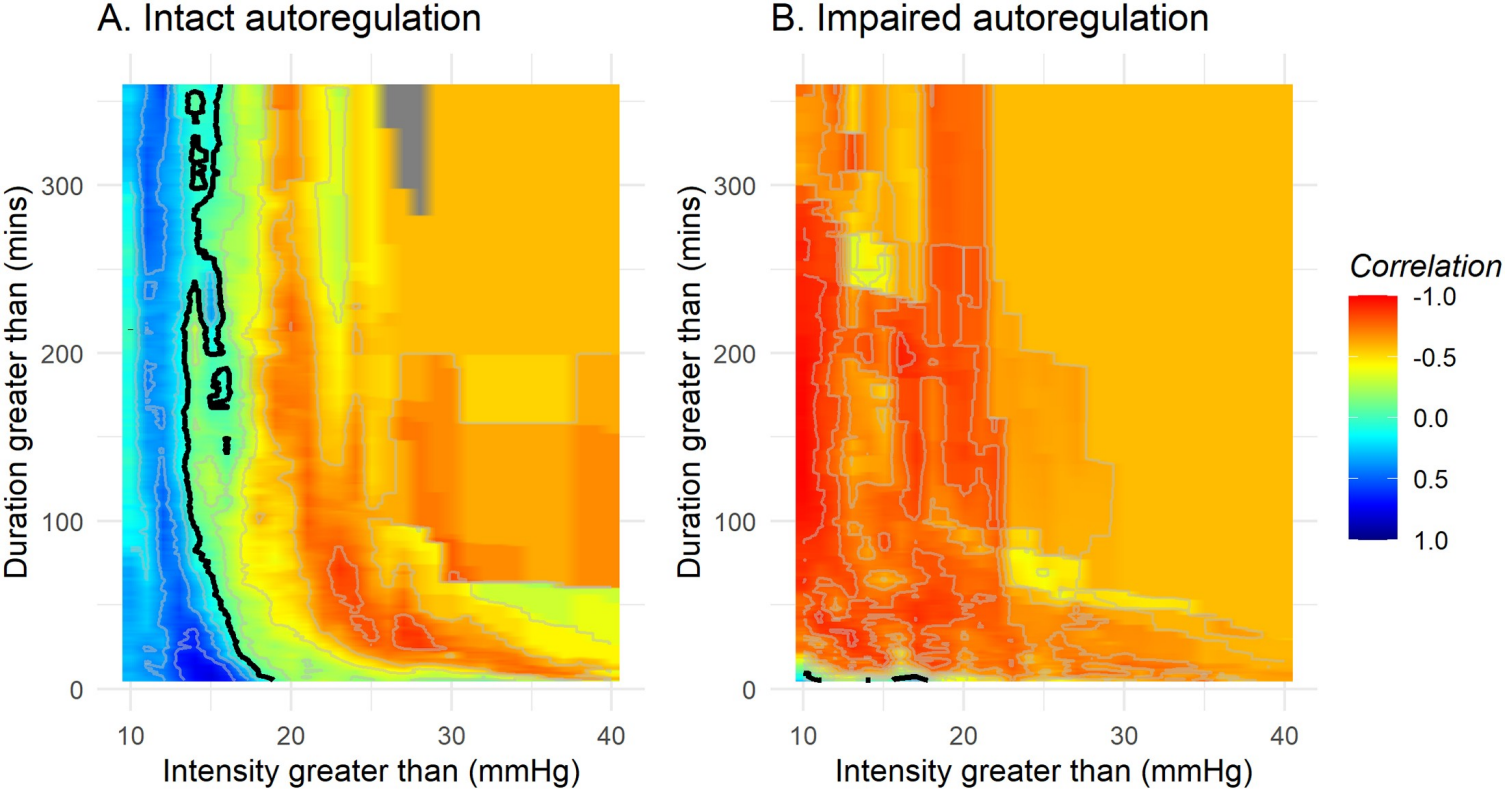
Correlation between number of events above thresholds of intracranial pressure intensity and duration and outcome, stratified by cerebral autoregulatory status. Orange / red areas indicates areas where ICP levels and event durations are associated with worse outcomes. The transition line, i.e. where there is no correlation between number of events and outcome, is drawn in black. All patients contribute some data to both plots, the degree however depending on the extent of their intact vs. impaired autoregulation. A) Intact autoregulation (mean PRx <= 0.3), B) Impaired autoregulation (mean PRx > 0.3).

**Fig 5:**
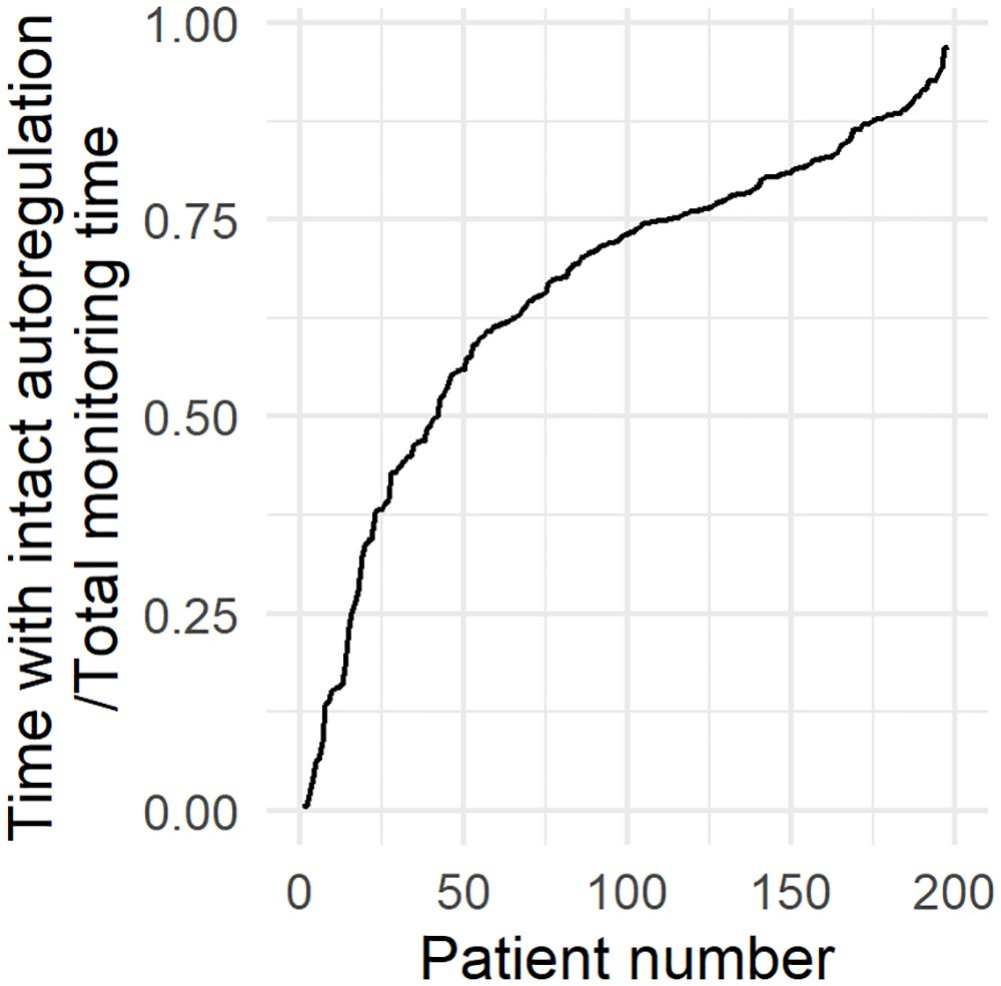
Fraction of monitoring time with intact autoregulation, per patient. All patients had both episodes of impaired and intact autoregulation, but to different extents.

To investigate the impact of cerebral autoregulation status on tolerability of ICP events, all events were stratified according to either intact (mean PRx <= 0.3) or impaired (mean PRx > 0.3) autoregulation, Fig 4A and 4B. All patients had, to different extents, both events with intact and impaired autoregulation, Fig 5, and 24.9% of the total monitoring time had a mean PRx > 0.3, indicating impaired autoregulation. In case of impaired autoregulation (Fig 4B), no threshold for tolerable ICP intensities and durations could be found.

In the univariable regression analysis, the time spent above the transition line was a statistically significant predictor of both unfavourable outcome and 6-month mortality, OR = 2.24 (95% CI 1.02–4.99, *p* = 0.046) and OR = 4.18 (95% CI 1.64–11.16, *p* = 0.003). When adjusted for the IMPACT core variables and maximum daily TIL, time above the transition line remained statistically significantly associated with mortality OR = 3.56 (95% CI 1.14–11.74, *p* = 0.032), but not with unfavourable outcome OR = 1.37 (95% CI 0.51–3.76, *p* = 0.533). A full summary of the regression is presented in Table C in S2 Appendix.

### Pressure and time dose of ICP

**Fig 6:**
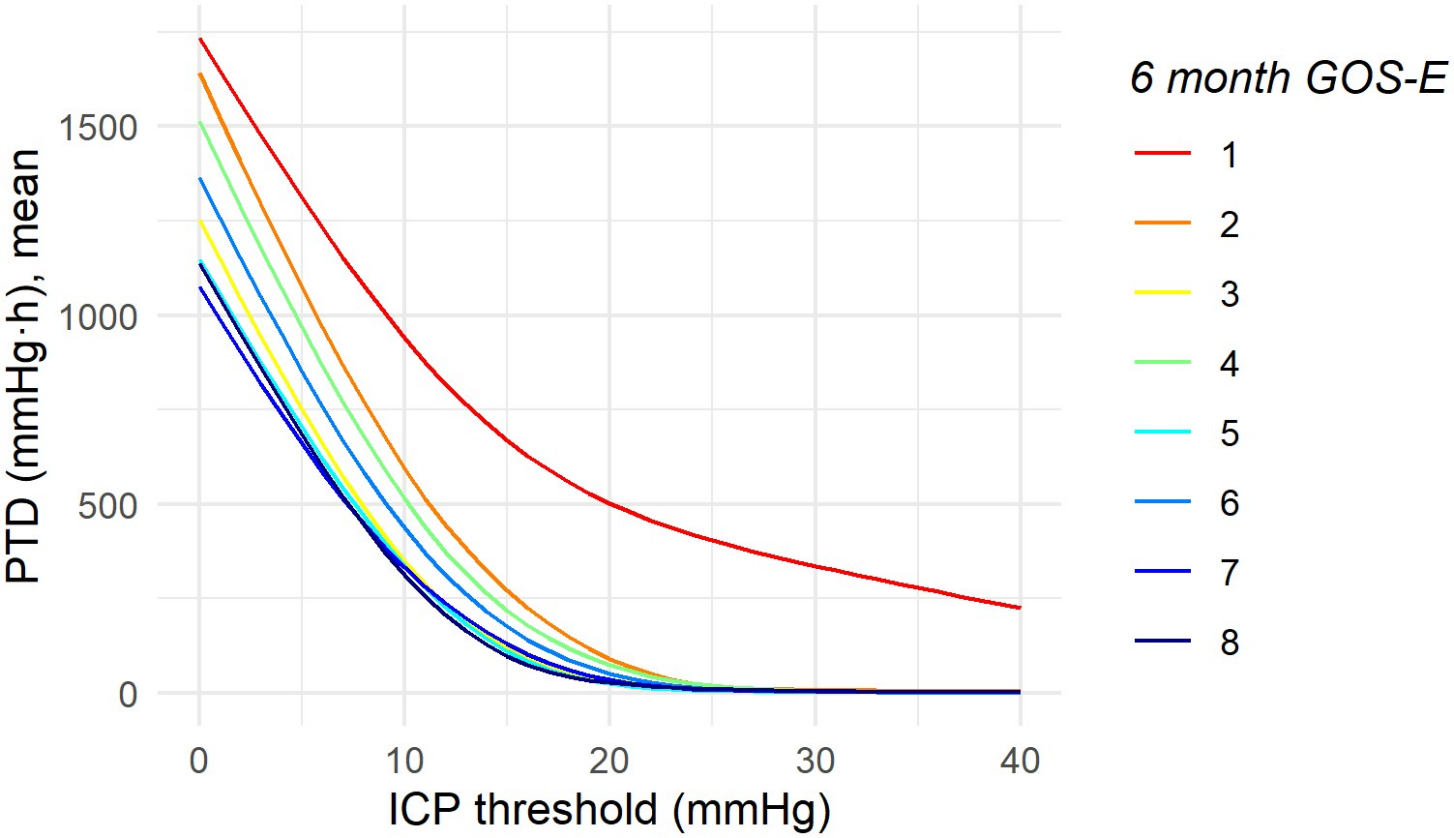
Total pressure and time dose (PTD) above ICP thresholds stratified by outcome at 6 months. The group mean PTD was higher for patients who had died within 6 months post injury, while the mean doses were similar for GOS-E 3 to 8 outcomes.

**Fig 7:**
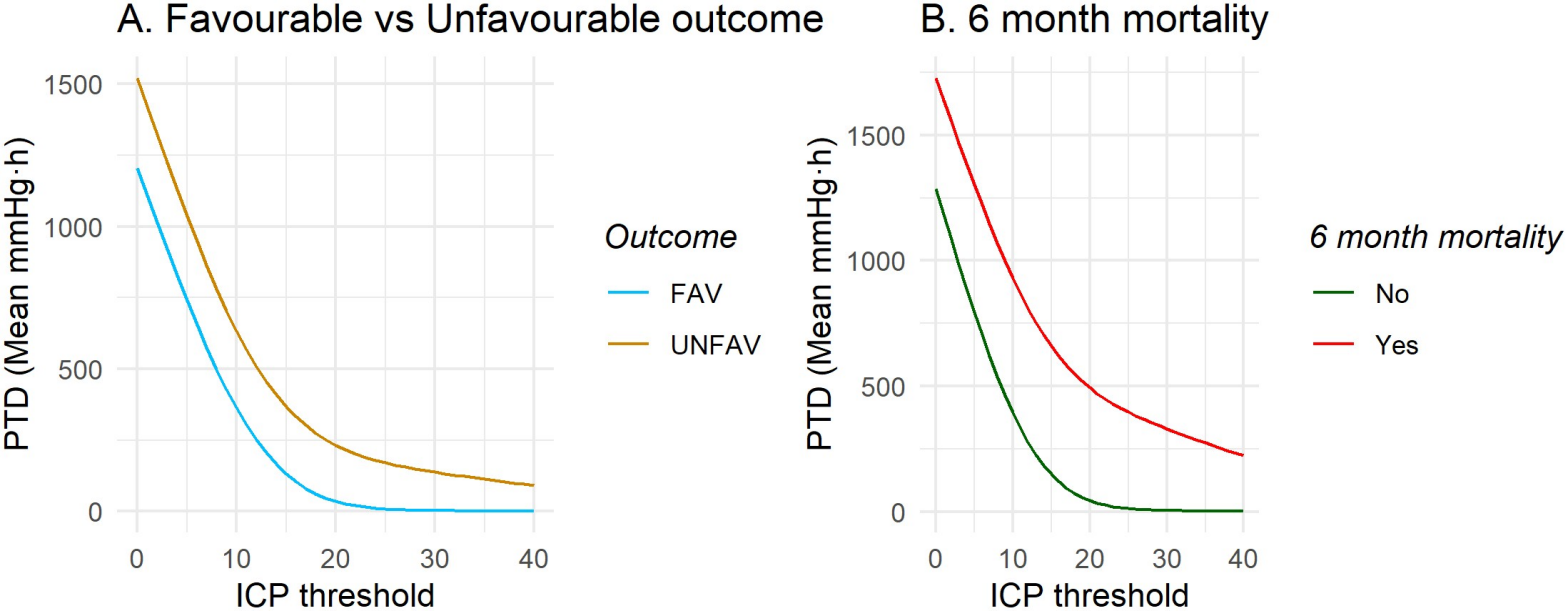
Group mean PTD (mmHg·h) above thresholds of ICP, median for A) favourable vs unfavourable outcome, B) dead vs alive at 6 months post injury.

The mean PTD above thresholds of 0 to 40 mmHg are presented for each category of GOS-E in Fig 6. Patients with unfavourable outcome had a significantly higher mean PTD above 20 and 25 mmHg compared to patients with favourable outcome, Fig 7A and Table 2, with PTD20 being 232.9 (± 750.8) vs 35.1 (± 64.5) mmHg·h respectively (*p* = 0.014).

**Table 2:** Group mean PTD (mmHg·h) above thresholds of ICP.

PTD	Favourable outcome	Unfavourable outcome	p	Alive at 6 months	Dead at 6 months	p
**0**	1205.4 (±632.4)	1519.9 (±1118.9)	0.160	1285.8 (±649.8)	1725.9 (±1547.3)	0.080
**10**	363.7 (±335)	633.3 (±956.6)	0.236	394.0 (±372.1)	930.8 (±1368.3)	0.004
**15**	132 (±175.6)	367.8 (±853.7)	0.173	148.8 (±210.1)	658.2 (±1255.7)	0.003
**20**	35.1 (±64.5)	232.9 (±750.8)	0.014	43.8 (±84.7)	493.4 (±1125.6)	0.001
**25**	8.7 (±16.3)	170.6 (±659.4)	0.017	11.9 (±24.7)	396.3 (±994.9)	0.003
**30**	3.2 (±6.2)	137.9 (±572.6)	0.071	4.5 (±10.6)	329.9 (±866.1)	0.003

On average, patients who died within 6 months post injury had a statistically significantly higher PTD above all ICP thresholds of 10 mmHg and above compared to survivors, Fig 7B and Table 2. The mean PTD above 20 mmHg was 493.4 (± 1125.6) vs 43.8 (± 84.7) mmHg·h (*p* = 0.004).

**Fig 8:**
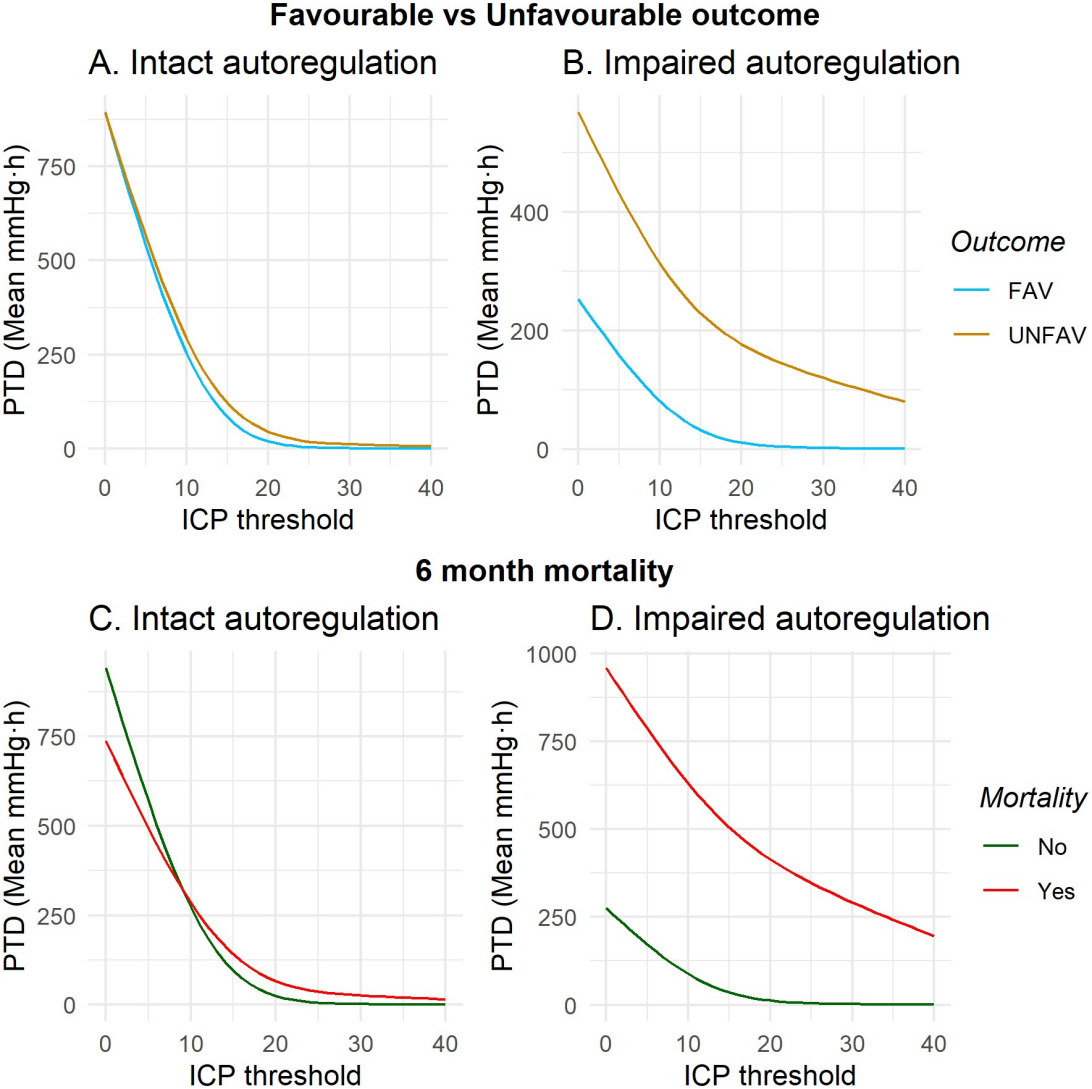
A) Intact vs B) impaired autoregulation, mean PTD for favourable and unfavourable outcome, C) Intact vs D) Impaired autoregulation for 6 month mortality.

PTD was also calculated separately for periods with intact (PTDintact) and impaired (PTDimpaired) autoregulation, Fig 8, Tables 3 and 4. There was no significant difference in mean PTD between favourable and unfavourable outcome at any ICP threshold when stratified by intact or impaired autoregulation, Fig 8A and Table 3.

**Table 3:** Group mean PTD (mmHg·h) of intact or impaired autoregulation above thresholds of ICP, favourable vs unfavourable outcome.

	Intact autoregulation			Impaired autoregulation		
PTD	Favorable Outcome	Unfavorable outcome	p	Favorable Outcome	Unfavorable outcome	p
**0**	893.4 (±531)	892.5 (±590.3)	0.870	252.6 (±171.9)	568.2 (±1009.9)	0.030
**10**	253.4 (±264)	291.9 (±328.7)	0.900	80.7 (±74.1)	312.9 (±847.5)	0.077
**15**	84 (±132.2)	121.9 (±203.3)	0.788	32.1 (±36.3)	228.7 (±766.8)	0.073
**20**	19.8 (±50.4)	45.6 (±113.9)	0.222	10.8 (±14.4)	177.2 (±684.2)	0.031
**25**	4.6 (±12.4)	19.4 (±79.5)	0.090	4 (±6.5)	144.3 (±603.2)	0.068
**30**	1.5 (±3.6)	12.1 (±65.6)	0.248	1.7 (±3.5)	120 (±524.8)	0.081

**Table 4:** Group mean PTD (mmHg·h) of intact or impaired autoregulation above thresholds of ICP, Dead or alive at 6 months, intact and impaired autoregulation.

	Intact autoregulation			Impaired autoregulation		
PTD	Alive at 6 months	Dead at 6 months	p	Alive at 6 months	Dead at 6 months	p
**0**	941 (±553.1)	738.1 (±581.3)	0.020	275.1 (±189.5)	959.2 (±1491.3)	< 0.001
**10**	272.8 (±297.5)	285.9 (±325)	0.760	88 (±82.4)	630.4 (±1269)	0.002
**15**	95.2 (±162.6)	141.5 (±218.4)	0.327	35.8 (±44)	505.3 (±1155.2)	0.001
**20**	25 (±64.5)	66.7 (±150.6)	0.037	12.8 (±20.1)	414.9 (±1035.6)	< 0.001
**25**	5.9 (±16.8)	36.8 (±121)	0.021	4.9 (±9.6)	347.4 (±915.7)	0.001
**30**	1.9 (±5.6)	26.3 (±101.8)	0.027	2.2 (±5.7)	292 (±798.5)	0.006

Additionally, autoregulation stratified mortality cohorts showed similar patterns to that of unstratified mortality but with a generally stronger association towards outcome. PTDintact and PTDimpaired stratified cohorts were significant related to mortality above all thresholds of ICP, Fig 8C and 8D and Table 4. The mean PTD of intact autoregulation above 20 mmHg was 66.7 (± 150.6) vs 25.0 (± 64.5) mmHg·h for non-survivors and survivors, respectively (*p* = 0.0037), and mean PTDimpaired above 20 mmHg was 414.9 (± 1035.6) and 12.8 (± 20.1), respectively (*p*<0.001).

When adjusted for the IMPACT core variables (age, GCS motor score at baseline and pupil reactivity) and maximum daily TIL, PTD was not an independent predictor of favourable outcome, OR = 1.0 (95% CI 0.99–1.00, *p* = 0.390), but still a significant predictor for 6 month mortality, OR = 1.0 (95% CI 1.00–1.01, *p* = 0.012), Table D in S2 Appendix. Neither PTDintact nor PTDimpaired were retained as significant predictors of 6-month mortality in a multivariable regression model, OR = 1.00 (95% CI 0.99–1.01, *p* = 0.238) and 1.02 (1.00–1.02, *p* = 0.236), respectively.

## Discussion

In this study we investigate the relationship between time-dependent ICP insults and outcome. We confirm findings of the Insult intensity/duration plot methodology, albeit finding lower acceptable ICP levels, in a new multicentre cohort and also investigate simpler pressure-time-dose measures. Importantly, we also introduce a novel bootstrap methodology to assess the certainty/uncertainty of the transition line of the correlation plot, above which there is an increased correlation between number of events and worse outcome. We believe this is a necessary extension to the insult intensity plots in order to interpret them with confidence. Additionally, we investigate relations of potential ICP vulnerability during periods of intact and impaired autoregulation suggesting that safe ICP levels may vary depending on autoregulatory status.

The overall pattern concerning association of ICP events and worse outcome in our study is similar to previously published results: However, we find lower limits for acceptable ICPs. In contrast to ICP thresholds, there is a higher degree of uncertainty in what duration of insult is associated with harm; the length of ICP events that are associated with worse outcome were more variable and population dependent. As indicated in Fig 3B, ICP levels above 18 ±4 mmHg for five minutes or longer are associated with worse outcome whereas another threshold at 13 mmHg with a wide time uncertainty is seen.

Above the +2 SD line there is a strong association between number of events at all levels of intensity and duration of ICP and worse outcome, and the time spent in this zone is strongly correlated both to 6-month mortality and unfavourable outcome. It can also be concluded that it is fairly certain that events to the right of the white line (mean transition line +2 SD) are associated with worse outcome. This corresponds to ICP events above 22 mmHg longer than 5 minutes and above 16 mmHg for longer than 60 minutes. As a comparison, previously suggested cut-offs from Güiza et al are 35 mmHg for 5 minutes or 20 mmHg for 37 minutes [15] and from Donnelly et al an ICP of 20 mmHg for longer than 13 minutes [16]. However, in both cases, correlations between 5-level GOS (rather than GOS-E as in this work) and ICP events were analysed. The first mentioned cohort was similar to ours with respect to age, admission GCS and cerebrovascular reactivity status, however the CENTER-TBI high-resolution cohort had a worse outcome at the group level. This may be attributable to numerous possible factors and could cause a general shift in our curves. To investigate if the difference in results were not due to using different outcome measures (GOS vs. GOS-E), we reproduced the same analysis with GOS as an outcome measure, with almost no difference in result (S3 Fig). It is important to mention that these results assume a linear relationship between number of events above thresholds and GOS-E score, which might be an inappropriate approximation and needs to be explored further. In summary, we find that although the pattern is similar between cohorts, absolute levels differ, supporting our effort to investigate certainty/uncertainty on regions of the map before defining or suggesting generalized cut-offs.

As with any observational analysis, the question is whether our results represent causation (i.e. if reducing ICP to lower insult levels in a timely way could affect outcome) or simply association, and the methods used in this study cannot distinguish between the two. However, it is biologically plausible that short periods of higher levels of ICP could be causally related to outcome although perhaps identified thresholds for longer time periods might represent associations at a cohort level as uncertainty is higher along this direction of the map.

The association between ICP intensity / duration and outcome are likely to be affected by any treatment directed at lowering of ICP. An attempt to adjust for these factors was done by including a measure of therapy intensity level in a multivariable regression model of time in red-orange zone and its association on outcome. This analysis suggested that the time above the transition line is a statistically significant predictor for death but not for unfavourable outcome.

Decompressive craniectomy (DC) is an intervention most likely affecting intracranial pressure levels as well as tolerability and reactivity. In the analysis, the 53 patients in our cohort who underwent DC were included. Including these patients might be regarded a limitation, particularly when previous studies have been inconsistent in whether PRx is affected or not [28, 29]. However, it is biologically plausible that ICP elevations are harmful to the brain per se, no matter whether DC has been performed or not. A separate analysis of this group alone could not be performed due to limited sample size and a seemingly greater inter-individual variation of ICP tolerability. A sensitivity analysis, excluding these patients is presented in S2 Fig yielded qualitatively similar results.

Broadly speaking, although our results suggest a somewhat lower threshold of ICP elevations our results are not dissimilar to both the BTF recommendations (ICP target below 22 mmHg [8]) and European neurointensive care practice (ICP 20 mmHg [4]) especially if bearing in mind that the error of measurement for ICP measurement is of the order of 1.5 mmHg [30].

Importantly, we also demonstrate that ICP tolerability appears highly dependent on cerebrovascular reactivity, Fig 4, and there appears to be no threshold for tolerable ICP during periods of disrupted autoregulation. In the case of intact autoregulation, our results suggest that an ICP above 19 mmHg for 5 minutes or longer or 15 mmHg for 50 minutes or longer was strongly associated with worse outcome. The finding that ICP tolerability may be dependent on cerebral autoregulation status is in line with previous studies which have suggested that autoregulation status or individualized ICP thresholds derived from autoregulation status seem to better predict outcome than fixed ICP levels [9, 31]. A similar pattern was also found by Güiza [15]. Further, it is biologically plausible that in the absence of intact autoregulation, the brain is left vulnerable and unable to compensate for global and regional changes in cerebral perfusion over time. Attempts to determine individual baseline ICPs by correlating the PRx to ICP to identify individualized ICP thresholds has been done by both Lazaridis et al and Zeiler et al [9, 31], an approach that needs to be further investigated in future studies. Several recent publications have also pointed at cerebrovascular reactivity being more important than fixed ICP thresholds in limiting secondary injuries [32–34]. It has also been suggested that it is the cerebral perfusion pressure (CPP) rather than the ICP that represent the true burden of secondary insult. The purpose of this study, however, was to investigate the impact of ICP, and the impact of treatments to optimise care (e.g. targeting CPPopt [35]) still need to be explored.

Our study and previous studies indicate that avoiding ICP peaks above 20 mmHg appears justified in aggregate across all patients, however during periods of autoregulatory loss, no safe limit can be identified.

### The role of pressure times time dose

The PTD may be an additional, simple, measure of ICP tolerability and has been suggested as a predictor for mortality and unfavourable outcome by several authors, especially doses of ICP 20 mmHg and above. However, methodology and choice of threshold for PTD has been varied between studies, making comparison difficult [12–14, 36]. As seen in Fig 6, we also identify a relationship between higher PTD and worse outcome. A statistical difference was identified between a PTD above 20 mmHg and unfavourable outcome, and that of PTD above ICP 10 mmHg and mortality. This may not represent absolute levels of ICP but a general association that is merely stronger in relation to mortality vs. favourable/unfavourable outcome. However, an association that, when adjusted for IMPACT core variables and TIL, remained significant for mortality but not unfavourable outcome (S2 Appendix Table B). Care is required in the final interpretation of PTD cut-off levels as ICP vulnerability might be expected to change over time with changing pathophysiology, as well as the individual metabolic states of the brain, and a next step would be to investigate the temporal evolution of ICP and ICP vulnerability.

Our results differ from previously reported PTD, which may reflect differences in the details of the methods used in its calculation with time windows from 24 hours up to total monitoring time as well as different time resolutions of ICP measurements. There is no choice that is clearly superior as a description of the ICP secondary insult burden. Nevertheless, despite these discrepancies, a relationship between PTD and outcome appears robust across studies.

In summary, we identify a clear relationship between high doses of ICP and mortality but not for favourable/unfavourable outcome. However, we also identify clear correlations between number of events above thresholds of ICP intensity, even with short durations, and worse outcome. In aggregate this could suggest that peaks of ICP elevation might in themselves be harmful. We hypothesize, given the greater uncertainty in the time dimension, that short periods of raised ICP may be causal of injury and long periods of moderately high ICP may be association with injury severity. If so, ICP variability may also be related to outcome and worth further future investigation.

### Advantages, limitations and future directions

A major strength of this study is its multi-center design where more than 220 patients from 20 sites across Europe were included. This, in combination with the applied bootstrapping technique minimizes potential confounding effects of site-specific treatments of severe TBI.

Some limitations must be noted. It is important to stress that the relationship which we have established between ICP magnitude and duration and outcome is associative, and none of the methods used in this paper can conclusively establish a causative relation. We have also made the assumption of a linear relationship, and cannot exclude that a non-linear model would make a better fit. Treatment of patients with severe TBI is complex, and the impact of treatments on outcome is not yet fully understood. Although we have adjusted for treatments in multivariable regression models, we have not been able to show if they act as confounders or are causative in the relationship of ICP and outcome. Furthermore, ICP vulnerability might change over time, something we have not taken into consideration in this study.

It would be of great interest to further investigate aspects of the temporal evolution of ICP and ICP vulnerability as well as to better establish potential causality in relation to outcome. Our results lead us to hypothesize that short periods of ICP elevations may be causal of injury and more extended periods of moderate elevation may be more associated to TBI severity. Future studies should focus on exploring the use of emerging techniques to evaluate causality with mathematical modelling and to investigate the impact of ICP variability on outcome.

## Conclusions

We have explored the relations of ICP towards outcome, employing several metrics of burden. We identify ICP limits and event durations associated with worse outcome, and importantly the uncertainty of such estimates. We find 18 mmHg to be the most probable safe ICP limit even for short durations. Given an uncertainty of ± 4 mmHg (± 2 SD), 22 mmHg can be identified as a limit that is with a high certainty related to worse outcome, and thus in concert with current BTF guidelines. However, it is lower than earlier event-duration plot studies. Although the adjusted ICP pressure time dose was strongly correlated to mortality, short periods of high ICP appear more confidently related to worse outcome than long periods of moderately high ICP leading us to hypothesize that the relation of burden towards outcome at lower ICP levels may be an association with injury severity, but shorter periods of elevated ICP may be more causative of injury. Additionally, we have found that ICP tolerability appears highly dependent on the cerebral autoregulation status where, in the case of impaired cerebrovascular reactivity, no safe ICP levels could be identified, suggesting that safe limits may need to be related to current autoregulatory status in the future.

## Supporting information

S1 Appendix List of ethical approvals for sites included in the high-resolution CENTER-TBI sub-study. (XLSX)

S2 Appendix Supplementary Tables. (DOCX)

S1 Fig Correlation between number of events above thresholds of intracranial pressure and duration and outcome (GOS-E score), ten illustrative bootstraps with replacement, sample size 209. The black line represents the transition line, above which there is a correlation between more events and worse outcome. As seen, the shape and values of the transition line is dependent on the patient selection.
(TIF)

S2 Fig Correlation between number of events above thresholds of intracranial pressure and duration and outcome (GOS-E score). A. All patients. B. All patients who has not undergone decompressive craniectomy. C. All patients with other monitors than extra-ventricular drain. **D.** All patients who has not undergone decompressive craniectomy and do not have an extra-ventricular drain.
(TIF)

S3 Fig Correlation between number of events above thresholds of intracranial pressure and duration and outcome (GOS score). (TIF)

S4 Fig Distribution of monitoring time in days, stratified by 6 month mortality. (TIF)

S5 Fig Distribution of mean PTD above thresholds of ICP 0 to 30 stratified by 6 month mortality status. (TIF)

## References

[1] Maas AIR, Menon DK, Adelson PD, Andelic N, Bell MJ, Belli A, et al Traumatic brain injury: integrated approaches to improve prevention, clinical care, and research. Lancet Neurol. 2017;16: 987–1048. 10.1016/S1474-4422(17)30371-X 29122524

[2] Sorrentino E, Diedler J, Kasprowicz M, Budohoski KP, Haubrich C, Smielewski P, et al Critical Thresholds for Cerebrovascular Reactivity After Traumatic Brain Injury. Neurocrit Care. 2012;16: 258–266. 10.1007/s12028-011-9630-8 21964774

[3] Carney N, Totten AM, O’Reilly C, Ullman JS, Hawryluk GWJ, Bell MJ, et al Guidelines for the Management of Severe Traumatic Brain Injury, Fourth Edition. Neurosurgery. 2016 10.1227/NEU.0000000000001432 27654000

[4] Cnossen MC, Huijben JA, Van Der Jagt M, Volovici V, Van Essen T, Polinder S, et al Variation in monitoring and treatment policies for intracranial hypertension in traumatic brain injury: a survey in 66 neurotrauma centers participating in the CENTER-TBI study. Crit Care. 2017;21. 10.1186/s13054-017-1816-9 PMC5586023 28874206

[5] Farahvar A, Gerber LM, Chiu Y-L, Carney N, Härtl R, Ghajar J. Increased mortality in patients with severe traumatic brain injury treated without intracranial pressure monitoring. J Neurosurg. 2012;117: 729–734. 10.3171/2012.7.JNS111816 22900846

[6] Chesnut RM, Temkin N, Carney N, Dikmen S, Rondina C, Videtta W, et al A Trial of Intracranial-Pressure Monitoring in Traumatic Brain Injury. n engl j med. 2012;36726367: 2471–81. 10.1056/NEJMoa1207363 23234472 PMC3565432

[7] Shafi S, Diaz-Arrastia R, Madden C, Gentilello L. Intracranial Pressure Monitoring in Brain-Injured Patients is Associated With Worsening of Survival. J Trauma Inj Infect Crit Care. 2008;64: 335–340. 10.1097/TA.0b013e31815dd017 18301195

[8] Aiolfi A, Elizabeth Benjamin B, Desmond Khor B, Kenji Inaba B, Lam L, Demetrios Demetriades B. Brain Trauma Foundation Guidelines for Intracranial Pressure Monitoring: Compliance and Effect on Outcome. World J Surg. 2017;41 10.1007/s00268-017-3898-6 28188356

[9] Lazaridis C, Desantis SM, Smielewski P, Menon DK, Hutchinson P, Pickard JD, et al Patient-specific thresholds of intracranial pressure in severe traumatic brain injury: Clinical article. J Neurosurg. 2014;120: 890–900. 10.3171/2014.1.JNS131292 24506248

[10] Zanier ER, Ortolano F, Ghisoni L, Colombo A, Losappio S, Stocchetti N. Intracranial pressure monitoring in intensive care: Clinical advantages of a computerized system over manual recording. Crit Care. 2007;11: 1–6. 10.1186/cc5155 17233895 PMC2151894

[11] Hemphill JC, Barton CW, Morabito D, Manley GT. Influence of data resolution and interpolation method on assessment of secondary brain insults in neurocritical care. Physiol Meas. 2005;26: 373–386. 10.1088/0967-3334/26/4/004 15886433

[12] Vik A, Nag T, Fredriksli OA, Skandsen T, Moen KG, Schirmer-Mikalsen K, et al Relationship of “dose” of intracranial hypertension to outcome in severe traumatic brain injury. J Neurosurg. 2008;109: 678–684. 10.3171/JNS/2008/109/10/0678 18826355

[13] Sheth KN, Stein DM, Aarabi B, Hu P, Kufera JA, Scalea TM, et al Intracranial pressure dose and outcome in traumatic brain injury. Neurocrit Care. 2013;18: 26–32. 10.1007/s12028-012-9780-3 23055087

[14] Magni F, Pozzi M, Rota M, Vargiolu A, Citerio G. High-Resolution Intracranial Pressure Burden and Outcome in Subarachnoid Hemorrhage. Stroke. 2015;46: 2464–2469. 10.1161/STROKEAHA.115.010219 26243224

[15] Güiza F, Depreitere B, Piper I, Citerio G, Chambers I, Jones PA, et al Visualizing the pressure and time burden of intracranial hypertension in adult and paediatric traumatic brain injury. Intensive Care Med. 2015;41: 1067–1076. 10.1007/s00134-015-3806-1 25894624

[16] Donnelly J, Güiza F, Depreitere B, Meyfroidt G, Czosnyka M, Smielewski P. Visualizing the pressure-time burden of elevated intracranial pressure after severe TBI—a retrospective confirmatory study. British Journal of Anaesthesia; 2020. 10.1016/j.bja.2020.09.018 33183738

[17] Maas AIR, Menon DK, Steyerberg EW, Citerio G, Lecky F, Manley GT, et al Collaborative European NeuroTrauma Effectiveness Research in Traumatic Brain Injury (CENTER-TBI): A Prospective Longitudinal Observational Study. Neurosurgery. 2015;76: 67–80. 10.1227/NEU.0000000000000575 25525693

[18] Steyerberg EW, Wiegers E, Sewalt C, Buki A, Citerio G, De Keyser V, et al Case-mix, care pathways, and outcomes in patients with traumatic brain injury in CENTER-TBI: a European prospective, multicentre, longitudinal, cohort study. Lancet Neurol. 2019;18: 923–934. 10.1016/S1474-4422(19)30232-7 31526754

[19] Czosnyka M, Smielewski P, Kirkpatrick P, Laing RJ, Menon D, Pickard JD. Continuous assessment of the cerebral vasomotor reactivity in head injury. Neurosurgery. 1997;41: 11–19. 10.1097/00006123-199707000-00005 9218290

[20] Pressure reactivity index (PRx). 2019 [cited 19 Nov 2019]. http://cppopt.org/prx/

[21] CENTER-TBI. CENTER-TBI Ethical Approval. https://www.center-tbi.eu/project/ethical-approval

[22] Adams H, Donnelly J, Czosnyka M, Kolias AG, Helmy A, Menon DK, et al Temporal profile of intracranial pressure and cerebrovascular reactivity in severe traumatic brain injury and association with fatal outcome: An observational study. PLoS Med. 2017;14: e1002353 10.1371/journal.pmed.1002353 28742817 PMC5526498

[23] Donnelly J, Czosnyka M, Adams H, Robba C, Steiner LA, Cardim D, et al Individualizing Thresholds of Cerebral Perfusion Pressure Using Estimated Limits of Autoregulation. Crit Care Med. 2017;45: 1464–1471. 10.1097/CCM.0000000000002575 28816837 PMC5595234

[24] Zeiler FA, Donnelly J, Smielewski P, Menon DK, Hutchinson PJ, Czosnyka M. Critical Thresholds of Intracranial Pressure-Derived Continuous Cerebrovascular Reactivity Indices for Outcome Prediction in Noncraniectomized Patients with Traumatic Brain Injury. J Neurotrauma. 2018;35: 1107–1115. 10.1089/neu.2017.5472 29241396

[25] R Core Team. R: A language and environment for statistical computing. Vienna, Austria: R Foundation for Statistical Computing; 2018 https://www.r-project.org/

[26] Maas AIR, Harrison-Felix CL, Menon D, Adelson PD, Balkin T, Bullock R, et al Standardizing data collection in traumatic brain injury. J Neurotrauma. 2011;28: 177–87. 10.1089/neu.2010.1617 21162610 PMC3037806

[27] Steyerberg EW, Mushkudiani N, Perel P, Butcher I, Lu J, Mchugh GS, et al Predicting Outcome after Traumatic Brain Injury: Development and International Validation of Prognostic Scores Based on Admission Characteristics. 2008 [cited 24 May 2018]. 10.1371/journal.pmed.0050165 PMC2494563 18684008

[28] Zeiler FA, Aries M, Cabeleira M, Van Essen TA, Stocchetti N, Menon DK, et al Statistical Cerebrovascular Reactivity Signal Properties after Secondary Decompressive Craniectomy in Traumatic Brain Injury: A CENTER-TBI Pilot Analysis. J Neurotrauma. 2020;37: 1306–1314. 10.1089/neu.2019.6726 31950876 PMC7249464

[29] Timofeev I, Czosnyka M, Nortje J, Smielewski P, Kirkpatrick P, Gupta A, et al Effect of decompressive craniectomy on intracranial pressure and cerebrospinal compensation following traumatic brain injury. J Neurosurg. 2008;108: 66–73. 10.3171/JNS/2008/108/01/0066 18173312

[30] Zacchetti L, Magnoni S, Di Corte F, Zanier ER, Stocchetti N. Accuracy of intracranial pressure monitoring: Systematic review and meta-analysis. Crit Care. 2015;19: 1–8. 26627204 10.1186/s13054-015-1137-9 PMC4667503

[31] Zeiler FA, Ercole A, Cabeleira M, Beqiri E, Zoerle T, Carbonara M, et al Patient-specific ICP Epidemiologic Thresholds in Adult Traumatic Brain Injury. J Neurosurg Anesthesiol. 2019; Publish Ah: 1–11. 31219937 10.1097/ANA.0000000000000616

[32] Smith M, Maas AIR. An algorithm for patients with intracranial pressure monitoring: filling the gap between evidence and practice. Intensive Care Med. 2019 10.1007/s00134-019-05818-4 31616963

[33] Nourallah B, Zeiler FA, Calviello L, Smielewski P, Czosnyka M, Menon DK. Critical thresholds for intracranial pressure vary over time in non-craniectomised traumatic brain injury patients. Acta Neurochir (Wien). 2018;160: 1315–1324. 10.1007/s00701-018-3555-3 29732476 PMC5996002

[34] Helbok R, Meyfroidt G, Beer R. Intracranial pressure thresholds in severe traumatic brain injury: Con: The injured brain is not aware of ICP thresholds! Intensive Care Med. 2018;44: 1318–1320. 10.1007/s00134-018-5249-y 29978388

[35] Steiner LA, Czosnyka M, Piechnik SK, Smielewski P, Chatfield D, Menon DK, et al Continuous monitoring of cerebrovascular pressure reactivity allows determination of optimal cerebral perfusion pressure in patients with traumatic brain injury. Crit Care Med. 2002;30: 733–738. 10.1097/00003246-200204000-00002 11940737

[36] Kahraman S, Dutton RP, Hu P, Xiao Y, Aarabi B, Stein DM, et al Automated measurement of “pressure times time dose” of intracranial hypertension best predicts outcome after severe traumatic brain injury. J Trauma—Inj Infect Crit Care. 2010;69: 110–118. 10.1097/TA.0b013e3181c99853 20038855

