# Supplementary material for: Impact of duration and magnitude of raised intracranial pressure on outcome after severe traumatic brain injury: A CENTER-TBI high-resolution group study
Source: PLoS One. 2020 Dec 14;15(12):e0243427. doi: 10.1371/journal.pone.0243427 (PMC7735618; doi:10.1371/journal.pone.0243427)
Supplement: S2 Appendix — (DOCX) [file pone.0243427.s002.docx]

# Supplementary tables to Impact of Duration and Magnitude of Raised Intracranial Pressure and Outcome after Severe Traumatic Brain Injury: A CENTER-TBI High Resolution Group Study

| Feature | n available |
| --- | --- |
| Age | 227 |
| Sex | 227 |
| ASA Class | 217 |
| Cause of injury | 227 |
| ISS | 227 |
| GCS (best pre-hospital) motor score | 222 |
| GCS (best pre-hospital), total score | 214 |
| Pupillary reactivity (at baseline) | 209 |
| Hypoxia (pre-ICU admission) | 196 |
| Hypotension (pre-ICU admission) | 197 |
| CT characteristics | 203 |
| Rotterdam CT Score | 198 |
| Hypoxia (during hospital stay) | 225 |
| Hypotension (during hospital stay) | 225 |
| Type of ICP device | 227 |
| Decompressive craniectomy | 227 |
| Length of stay, days | 221 |
| Length of stay in ICU, days | 222 |
| Monitoring time, days | 227 |
| Mean ICP, mmHg | 227 |
| Mean body temperature, C | 227 |
| Mean CPP, mmHg | 227 |
| Sodium day 2 post injury | 207 |
| GOS-E at 6 months | 227 |

**S2 Table A:** Number of patients with data available

|  | Favourable vs unfavourable outcome | |  | 6 month mortality | |
| --- | --- | --- | --- | --- | --- |
| Variable | **OR, 95% CI** | **p** |  | **OR, 95% CI** | **p** |
| (Intercept) | 0.10 (0.02-0.41) | 0.002 |  | 0.004 (0.00-0.03) | < 0.001 |
| Time above transition line | 1.37 (0.51-3.76) | 0.533 |  | 3.56 (1.14-11.74) | 0.032 |
| Age | 1.05 (1.03-1.07) | < 0.001 |  | 1.06 (1.03-1.08) | < 0.001 |
| GCS Motor score (baseline) | 0.69 (0.57-0.83) | < 0.001 |  | 0.86 (0.70-1.05) | 0.141 |
| Pupil reactivity (baseline) | 1.61 (1.03-2.59) | 0.041 |  | 1.62 (1.02-2.58) | 0.042 |
| Maximum Daily TIL | 1.13 (1.06-1.22) | < 0.001 |  | 1.08 (1.01-1.16) | 0.025 |

**S2 Table B: Multivariable model of time above transition line.**

|  | Favourable vs unfavourable outcome | |  | 6 month mortality | |
| --- | --- | --- | --- | --- | --- |
| Variable | **OR, 95% CI** | **p** |  | **OR, 95% CI** | **p** |
| (Intercept) | 0.12 (0.03-0.49) | 0.004 |  | 0.01 (0.00-0.05) | < 0.001 |
| PTD above 20 mmHg | 1.00 (1.00-1.01) | 0.192 |  | 1.00 (1.00-1.01) | 0.012 |
| Age | 1.05 (1.03-1.07) | < 0.001 |  | 1.05 (1.03-1.08) | < 0.001 |
| GCS Motor score (baseline) | 0.70 (0.58-0.84) | < 0.001 |  | 0.89 (0.72-1.10) | 0.297 |
| Pupil reactivity (baseline) | 1.58 (1.00-2.54) | 0.054 |  | 1.55 (0.95-2.54) | 0.079 |
| Maximum Daily TIL | 1.12 (1.05-1.20) | 0.001 |  | 1.08 (1.00-1.16) | 0.047 |

**S2 Table C: Multivariable model of PTD above ICP 20 mmHg.**

|  | Favourable vs unfavourable outcome | |  | 6 month mortality | |
| --- | --- | --- | --- | --- | --- |
| Variable | **OR, 95% CI** | **p** |  | **OR, 95% CI** | **p** |
| (Intercept) | 0.13 (0.03-0.56) | 0.008 |  | 0.00 (0.00-0.04) | < 0.001 |
| PTDintact above 20 mmHg | 1.00 (0.99-1.00) | 0.390 |  | 1.00 (0.99-1.01) | 0.238 |
| Age | 1.04 (1.02-1.07) | < 0.001 |  | 1.06 (1.04-1.09) | < 0.001 |
| GCS Motor score (baseline) | 0.70 (0.57-0.85) | < 0.001 |  | 0.86 (0.68-1.07) | 0.173 |
| Pupil reactivity (baseline) | 1.40 (0.89-2.29) | 0.163 |  | 1.46 (0.87-2.45) | 0.148 |
| Maximum Daily TIL | 1.14 (1.06-1.23) | < 0.001 |  | 1.10 (1.02-1.88) | 0.011 |

**S2 Table D: Multivariable model of PTD of intact autoregulation above ICP 20 mmHg.**

| Variable | Favourable vs unfavourable outcome | |  | 6 month mortality | |
| --- | --- | --- | --- | --- | --- |
|  | **OR, 95% CI** | **p** |  | **OR, 95% CI** | **p** |
| (Intercept) | 0.14, (0.03-0.57) | 0.008 |  | 0.00, (0.00-0.03) | < 0.001 |
| PTDimpaired above 20 mmHg | 1.01, (1.00-1.03) | 0.285 |  | 1.01, (1.00-1.02) | 0.236 |
| Age | 1.04, (1.02-1.06) | < 0.001 |  | 1.06, (1.03-1.10) | < 0.001 |
| GCS Motor score (baseline) | 0.71, (0.58-0.86) | 0.001 |  | 0.89, (0.70-1.12) | 0.324 |
| Pupil reactivity (baseline) | 1.33, (0.83-2.19) | 0.245 |  | 1.33, (0.75-2.33) | 0.319 |
| Maximum Daily TIL | 1.14, (1.06-1.23) | 0.001 |  | 1.09, (1.01-1.18) | 0.029 |

**S2 Table E: Multivariable model of PTD of impaired autoregulation above ICP 20 mmHg.**
