# Supplementary figures and images for: Impact of duration and magnitude of raised intracranial pressure on outcome after severe traumatic brain injury: A CENTER-TBI high-resolution group study
Source: PLoS One. 2020 Dec 14;15(12):e0243427. doi: 10.1371/journal.pone.0243427 (PMC7735618; doi:10.1371/journal.pone.0243427)

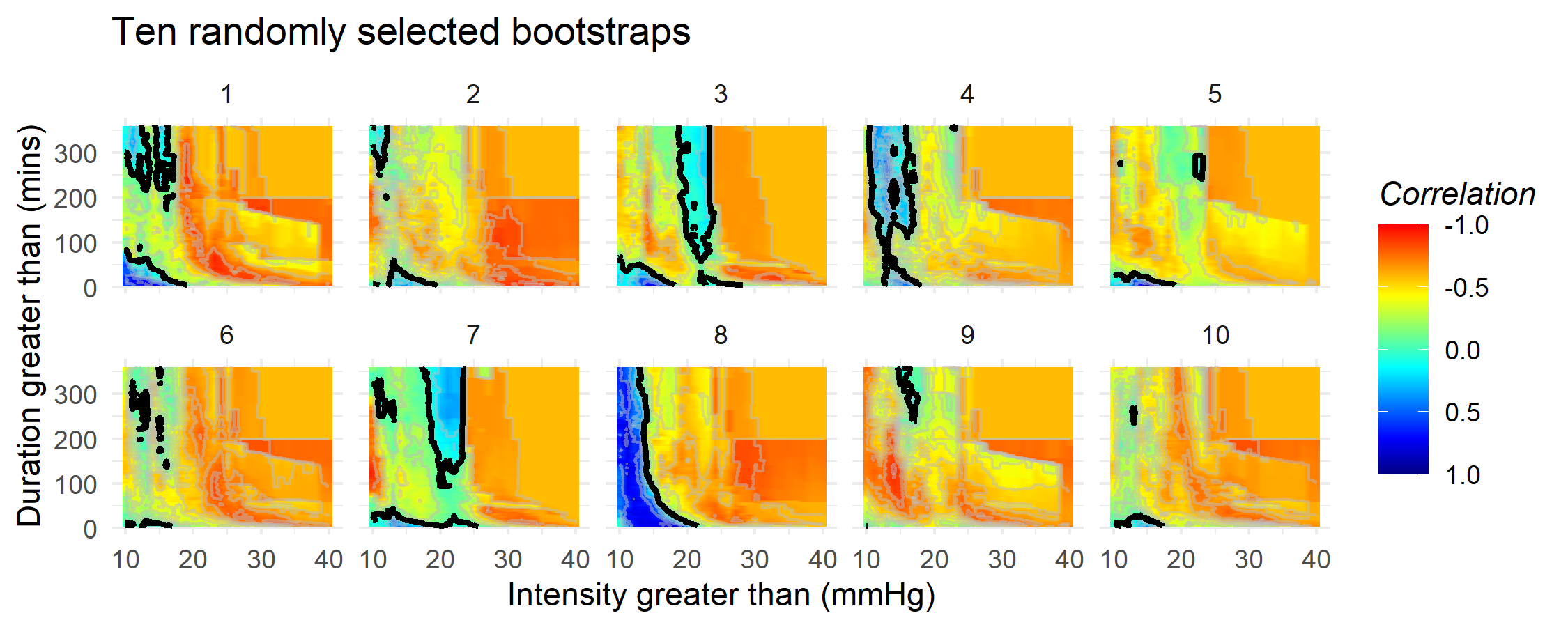

Supplement: S1 Fig — The black line represents the transition line, above which there is a correlation between more events and worse outcome. As seen, the shape and values of the transition line is dependent on the patient selection. (TIF) [file pone.0243427.s003.tif]

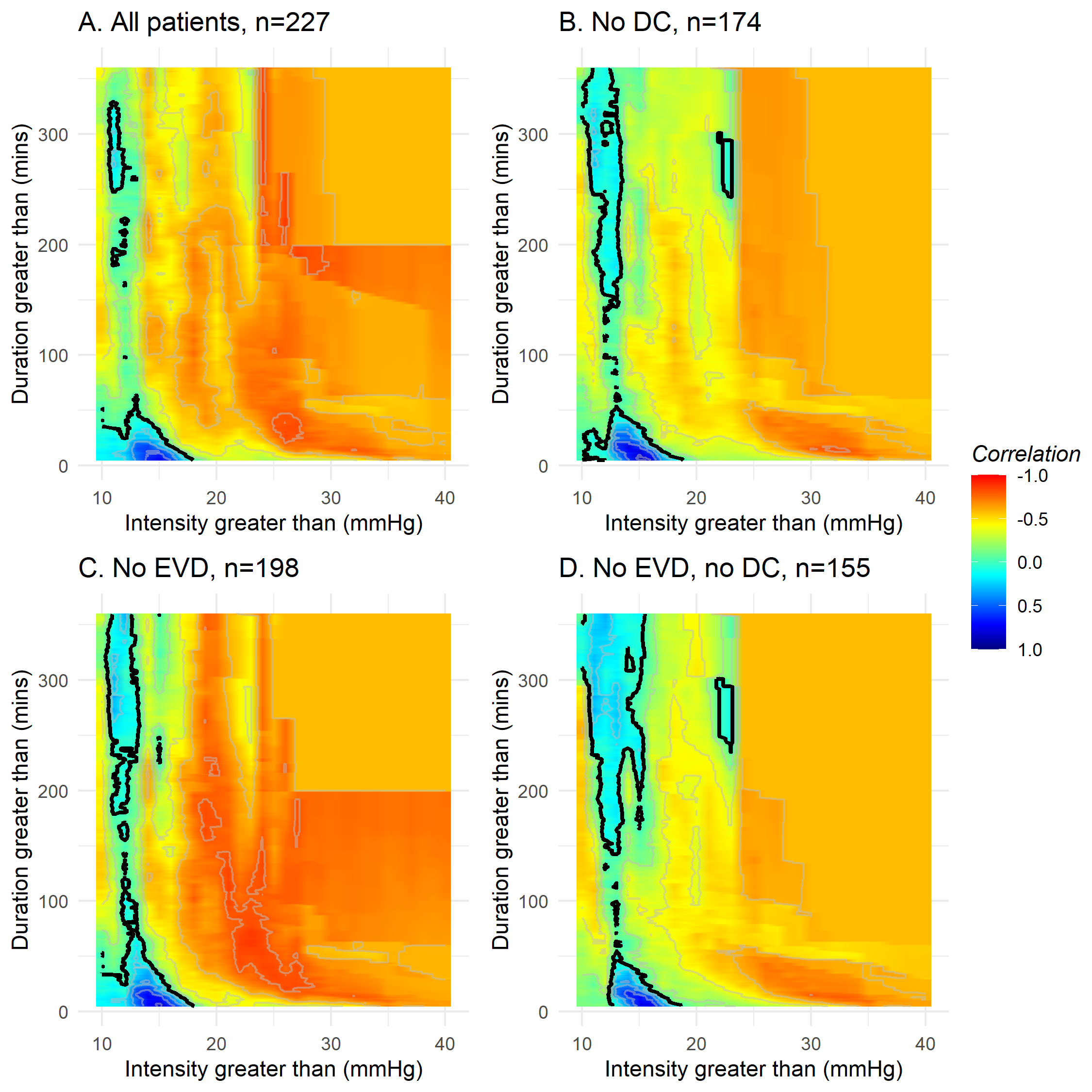

Supplement: S2 Fig — A. All patients. B. All patients who has not undergone decompressive craniectomy. C. All patients with other monitors than extra-ventricular drain. D. All patients who has not undergone decompressive craniectomy and do not have an extra-ventricular drain. (TIF) [file pone.0243427.s004.tif]

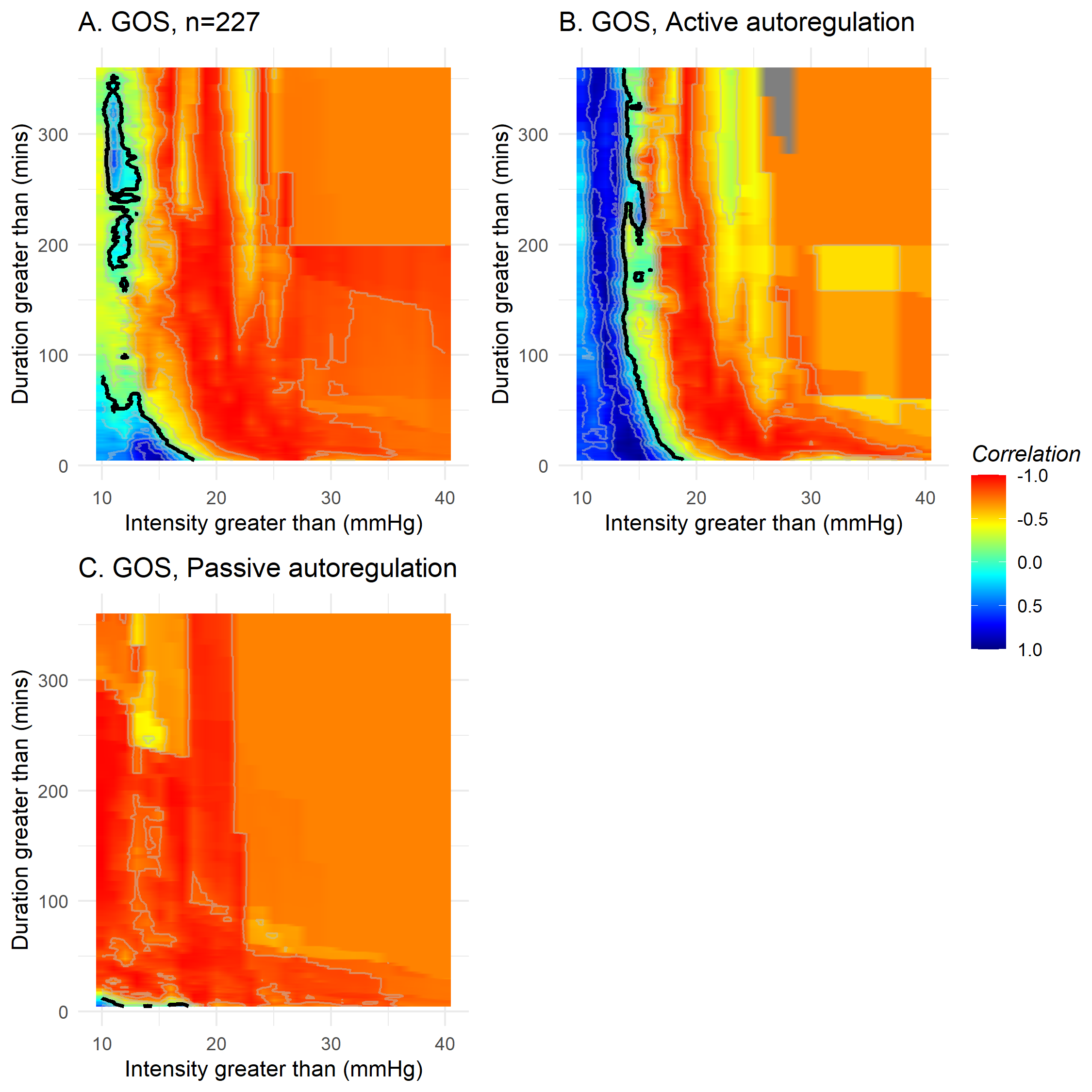

Supplement: S3 Fig — (TIF) [file pone.0243427.s005.tif]

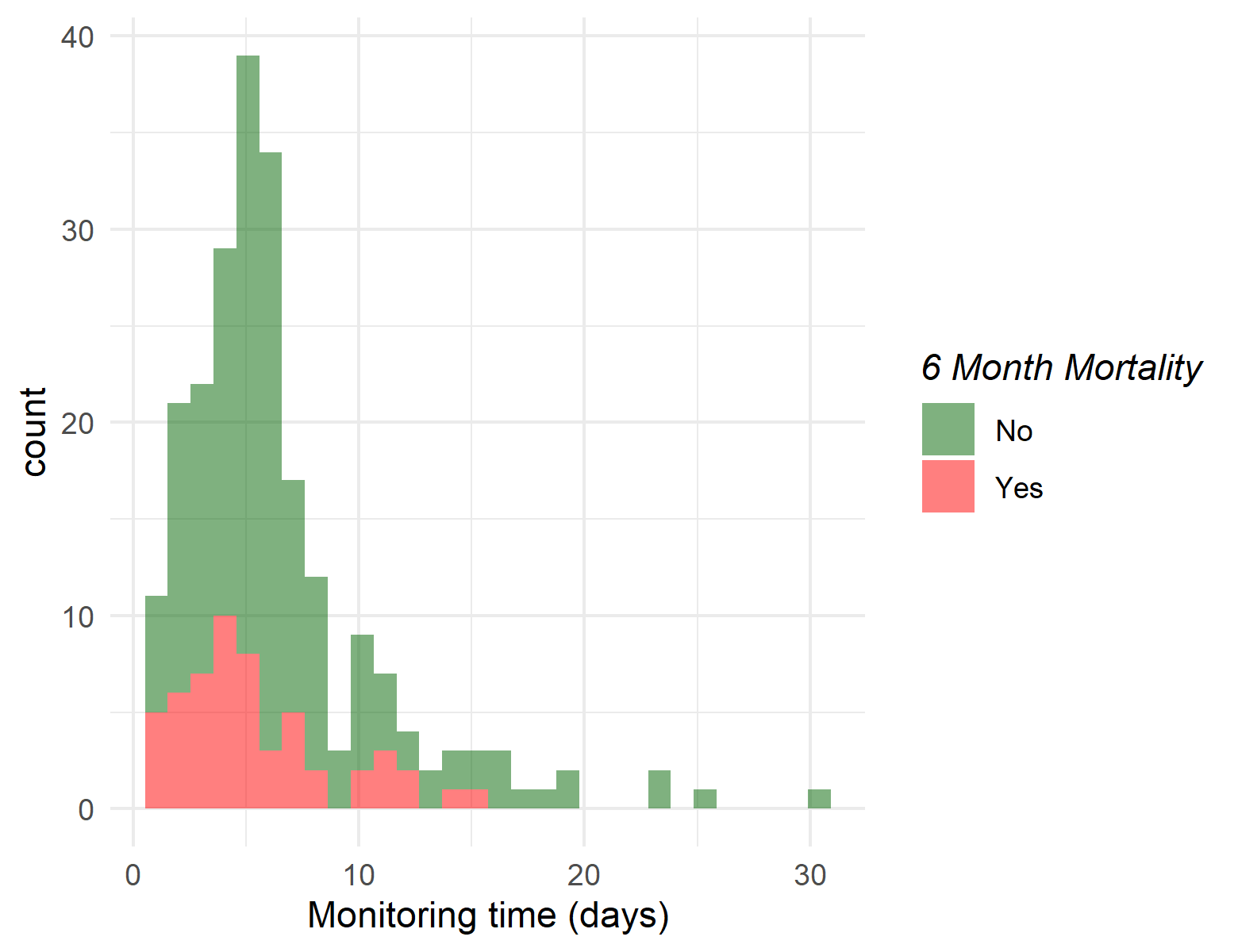

Supplement: S4 Fig — (TIF) [file pone.0243427.s006.tif]

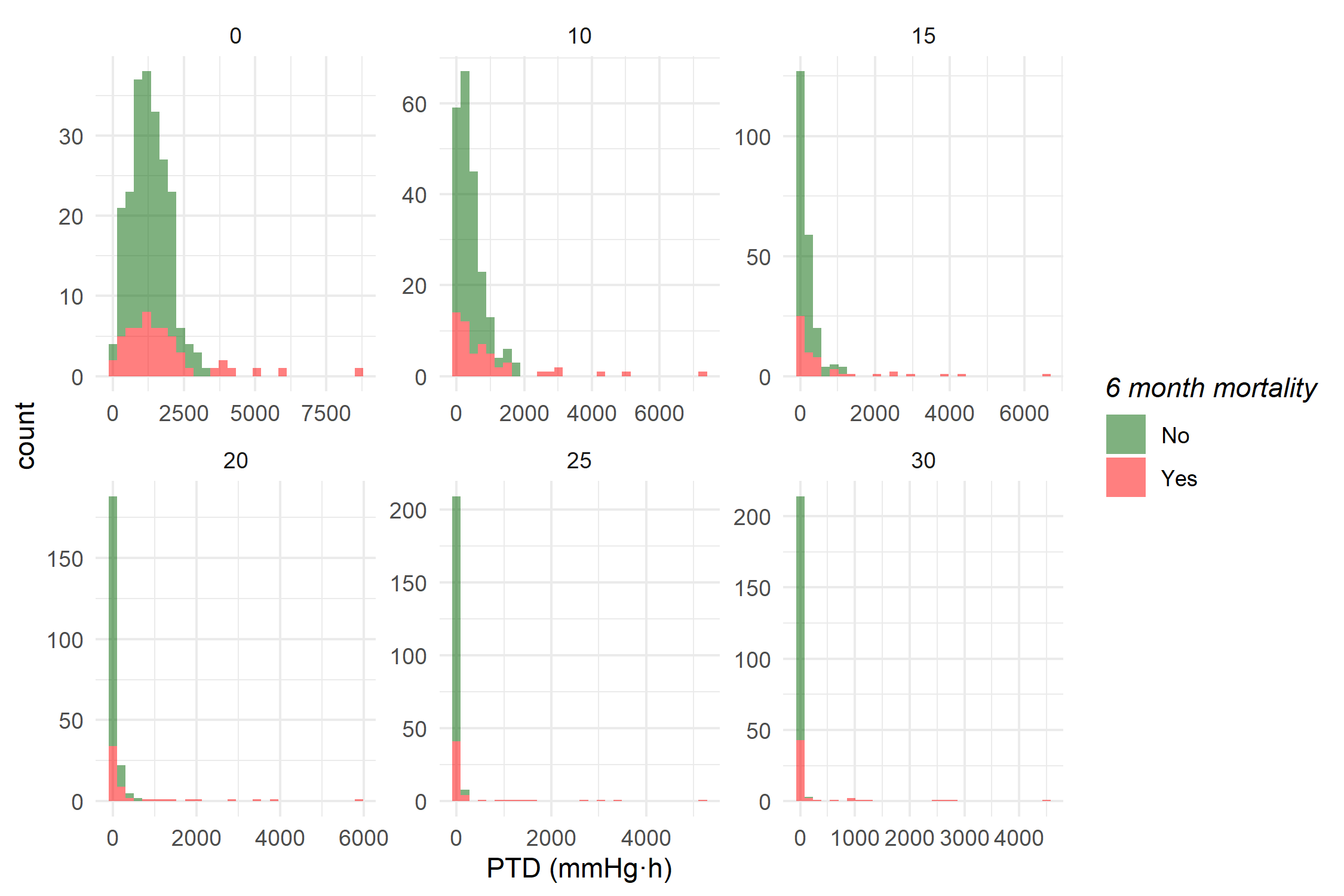

Supplement: S5 Fig — (TIF) [file pone.0243427.s007.tif]
