# Supplementary material for: Impact of duration and magnitude of raised intracranial pressure on outcome after severe traumatic brain injury: A CENTER-TBI high-resolution group study
Source: PLoS One. 2020 Dec 14;15(12):e0243427. doi: 10.1371/journal.pone.0243427 (PMC7735618; doi:10.1371/journal.pone.0243427)
Supplement: Attachment — Submitted filename: Response to Reviewers updated.docx [file pone.0243427.s008.docx]

*Dear Dr Kobeissy and the Reviewers,*

*Thank you for your encouraging words and quick reply, and for valuable comments. We have carefully considered all of them, and made changes to the manuscript accordingly, as can be seen in the attached manuscript files. Here follows replies to all your comments.*

**1. Please ensure that your manuscript meets PLOS ONE's style requirements, including those for *file naming.***

*Thank you for stating this. We have carefully gone through the manuscript and updated it to adhere to the style requirements. Please let us know if the manuscript should be further updated.*

**2. Please amend your current ethics statement to include the full name of the ethics committee/institutional review board(s) that approved your specific study.**

**Please provide additional details regarding participant consent. In the ethics statement in the Methods and online submission information, *please ensure that you have specified (1) whether consent was informed and (2) what type you obtained (for instance, written or verbal, and if verbal, how it was documented and witnessed).* If your study included minors, state whether you obtained consent from parents or guardians. If the need for consent was waived by the ethics committee, please include this information.**

*The ethics statement has been updated, in the methods section as well as in the online submission form, to include a reference to the ethical approvals for the sites in the high-resolution group, which recruited patients for this study. The type of consent has been clarified.*

*Modifications:*

“This study was approved by the CENTER-TBI management committee. The CENTER-TBI study was conducted in accordance with all relevant laws of the European Union if directly applicable or of direct effect and all relevant laws of the country where the Recruiting sites were located, including but not limited to, the relevant privacy and data protection laws and regulations (the “Privacy Law”), the relevant laws and regulations on the use of human materials, and all relevant guidance relating to clinical studies from time to time in force including, but not limited to, the ICH Harmonised Tripartite Guideline for Good Clinical Practice (CPMP/ICH/135/95) (“ICH GCP”) and the World Medical Association Declaration of Helsinki. Written or oral Informed Consent by the patients or next of kin was obtained, according to the local legislations, for all patients recruited in the Core Dataset of CENTER-TBI and documented in the electronic case report form. In case of oral consent, a written confirmation was requested.

Ethical approval was obtained for each recruiting site. The list of sites, Ethical Committees, approval numbers and approval dates are available online [21] and ethical approval numbers for sites having recruited patients to the high-resolution sub-study of CENTER-TBI is listed in S1 Appendix.”

**3. We note that you have indicated that data from this study are available upon request. PLOS only allows data to be available upon request if there are legal or ethical restrictions on sharing data publicly. For information on unacceptable data access restrictions, please see** [**http://journals.plos.org/plosone/s/data-availability#loc-unacceptable-data-access-restrictions**](http://journals.plos.org/plosone/s/data-availability#loc-unacceptable-data-access-restrictions)**.**

**In your revised cover letter, please address the following prompts:**

**a) If there are ethical or legal restrictions on sharing a de-identified data set, please explain them in detail (e.g., data contain potentially identifying or sensitive patient information) and who has imposed them (e.g., an ethics committee). Please also provide contact information for a data access committee, ethics committee, or other institutional body to which data requests may be sent.**

**b) If there are no restrictions, please upload the minimal anonymized data set necessary to replicate your study findings as either Supporting Information files or to a stable, public repository and provide us with the relevant URLs, DOIs, or accession numbers. Please see** [**http://www.bmj.com/content/340/bmj.c181.long**](http://www.bmj.com/content/340/bmj.c181.long) **for guidelines on how to de-identify and prepare clinical data for publication. For a list of acceptable repositories, please see** [**http://journals.plos.org/plosone/s/data-availability#loc-recommended-repositories**](http://journals.plos.org/plosone/s/data-availability#loc-recommended-repositories)**.**

**We will update your Data Availability statement on your behalf to reflect the information you provide.**

*It is not possible at this time point to share the data set freely due to the complexity of the data and a clear risk of identifying individual patients. However, if a legitimate scientific interest or need exists, it is possible to contact the CENTER-TBI steering committee for permission. Therefore, we have updated the data sharing paragraph to the following:*

“CENTER-TBI encourages data sharing, and there is a data sharing statement published: <https://center-tbi.eu/data/sharing>. Ethical and potential legal restrictions apply due to concerns regarding potential accidental re-identification of patients from such a complex and comprehensive dataset. Data will be available to researchers who provide a study proposal that is approved by the management committee to achieve the aims in the approved proposal. Proposals can be submitted online at <https://www.center-tbi.eu/data>. A request to access data to control analysis of a published article will be addressed in the same manner. A data access agreement is required and all access must comply with regulatory restrictions imposed on the original study.”

**4. We note that you received funding from a commercial source: [Integra LifeSciences Corporation]. Please provide an amended Competing Interests Statement that explicitly states this commercial funder, along with any other relevant declarations relating to employment, consultancy, patents, products in development, marketed products, etc.**

*Thank you for mentioning this. We would like to clarify Integra LifeSciences role in the study by changing the Competing Interests Statement as stated in the Revision Cover Letter.*

**5. One of the noted authors is a group or consortium [ENTER-TBI High Resolution ICU Sub-Study Participants and Investigators]. In addition to naming the author group, please list the individual authors and affiliations within this group in the acknowledgments section of your manuscript. Please also indicate clearly a lead author for this group along with a contact email address.**

*We have added the High resolution sub-study participants and investigators to the acknowledgement.*

**6. Please include a copy of Table 5 which you refer to in your text on page 15.**

*Thank you for finding this typo, the reference has been corrected to table 4.*

**Additional Editor Comments (if provided):

Dear Drs Åkerlund and Nelson,
this is a very nice work that has been evaluated by top researchers in the area of TBI.
i would request adding some sections related to limitations, shortcomings and future directions.**

*Thank you for your kind words, happy to hear that you appreciate our work. As requested, a section concerning limitations, advantages and future directions have been added to the discussion.*

**Reviewers comments**

**Reviewer #1:**

**1. show mmhg x hrs rather than using astrc * or use a centered dot.**
*Thank you for noting this. Asterices have been changed to centered dots.*

**2. Describe in text more detail about how autoregulation measures were assessed in simple terms**
*We have also added a short description on how autoregulation measurements were calculated and assessed, in the method section.*

**3. In discussion include a formal section on advantages and limitations...**
*In line with requests from reviewer #1 and Editor Dr Kobeissy, we have added a sub-section in the discussion section on advantages, limitations and future directions.*

*Once again, thank you for a quick and encouraging reply. We hope that you agree that our updates have improved the manuscript. Looking forward to your feedback soon.*

*Best wishes,*

*Cecilia Åkerlund and David Nelson*
